# The G Protein Estrogen Receptor (GPER) is involved in the resistance to the CDK4/6 inhibitor palbociclib in breast cancer

**DOI:** 10.1186/s13046-024-03096-7

**Published:** 2024-06-18

**Authors:** Marianna Talia, Francesca Cirillo, Domenica Scordamaglia, Marika Di Dio, Azzurra Zicarelli, Salvatore De Rosis, Anna Maria Miglietta, Carlo Capalbo, Ernestina Marianna De Francesco, Antonino Belfiore, Fedora Grande, Bruno Rizzuti, Maria Antonietta Occhiuzzi, Giancarlo Fortino, Antonella Guzzo, Gianluigi Greco, Marcello Maggiolini, Rosamaria Lappano

**Affiliations:** 1 Department of Pharmacy, Health and Nutritional Sciences, University of Calabria, Rende, 87036 Italy; 2 Breast and General Surgery Unit, Regional Hospital Cosenza, Cosenza, 87100 Italy; 3 Complex Operative Oncology Unit, Regional Hospital Cosenza, Cosenza, 87100 Italy; 4 Department of Medicine and Surgery, University of Enna Kore, Enna, 94100 Italy; 5 Department of Clinical and Experimental Medicine, University of Catania, Garibaldi-Nesima Hospital, Catania, 95122 Italy; 6 Department of Physics, CNR-NANOTEC, SS Rende (CS), University of Calabria, Rende, CS 87036 Italy; 7 Institute of Biocomputation and Physics of Complex Systems (BIFI), Joint Unit GBsC-CSIC-BIFI, University of Zaragoza, Zaragoza, 50018 Spain; 8 Department of Informatics, Modeling, Electronic, and System Engineering, University of Calabria, Rende, 87036 Italy; 9 Department of Mathematics and Computer Science, University of Calabria, Cosenza, Italy

**Keywords:** Palbociclib, Resistance, Breast cancer, Estrogen receptor, G protein-coupled estrogen receptor (GPER), Cancer-associated fibroblasts (CAFs)

## Abstract

**Background:**

The cyclin D1-cyclin dependent kinases (CDK)4/6 inhibitor palbociclib in combination with endocrine therapy shows remarkable efficacy in the management of estrogen receptor (ER)-positive and HER2-negative advanced breast cancer (BC). Nevertheless, resistance to palbociclib frequently arises, highlighting the need to identify new targets toward more comprehensive therapeutic strategies in BC patients.

**Methods:**

BC cell lines resistant to palbociclib were generated and used as a model system. Gene silencing techniques and overexpression experiments, real-time PCR, immunoblotting and chromatin immunoprecipitation studies as well as cell viability, colony and 3D spheroid formation assays served to evaluate the involvement of the G protein-coupled estrogen receptor (GPER) in the resistance to palbociclib in BC cells. Molecular docking simulations were also performed to investigate the potential interaction of palbociclib with GPER. Furthermore, BC cells co-cultured with cancer-associated fibroblasts (CAFs) isolated from mammary carcinoma, were used to investigate whether GPER signaling may contribute to functional cell interactions within the tumor microenvironment toward palbociclib resistance. Finally, by bioinformatics analyses and k-means clustering on clinical and expression data of large cohorts of BC patients, the clinical significance of novel mediators of palbociclib resistance was explored.

**Results:**

Dissecting the molecular events that characterize ER-positive BC cells resistant to palbociclib, the down-regulation of ERα along with the up-regulation of GPER were found. To evaluate the molecular events involved in the up-regulation of GPER, we determined that the epidermal growth factor receptor (EGFR) interacts with the promoter region of GPER and stimulates its expression toward BC cells resistance to palbociclib treatment. Adding further cues to these data, we ascertained that palbociclib does induce pro-inflammatory transcriptional events via GPER signaling in CAFs. Of note, by performing co-culture assays we demonstrated that GPER contributes to the reduced sensitivity to palbociclib also facilitating the functional interaction between BC cells and main components of the tumor microenvironment named CAFs.

**Conclusions:**

Overall, our results provide novel insights on the molecular events through which GPER may contribute to palbociclib resistance in BC cells. Additional investigations are warranted in order to assess whether targeting the GPER-mediated interactions between BC cells and CAFs may be useful in more comprehensive therapeutic approaches of BC resistant to palbociclib.

**Supplementary Information:**

The online version contains supplementary material available at 10.1186/s13046-024-03096-7.

## Background

Breast cancer (BC) is the most commonly diagnosed malignancy in women and it accounts for 31% of the female tumors worldwide [1]. The management of the different subtypes of BC is mainly determined by the expression of peculiar receptors, particularly the estrogen receptor (ER), the progesterone receptor (PR) and the human epidermal growth factor receptor 2 (HER2) [2]. Considering that approximately 70% of patients are characterized by ER-positive and HER2-negative BC, endocrine therapy is the mainstay of the therapeutic approach [3, 4]. Unfortunately, many patients display de novo or acquired resistance to endocrine therapy [5, 6], pointing out the need to fully dissect the molecular mechanisms involved in the resistance to endocrine therapy as well as to uncover alternate treatments [7].

The transmembrane G protein-coupled estrogen receptor (GPER) has been shown to mediate stimulatory effects elicited by estrogens, estrogen-like compounds and even antiestrogens in normal and malignant cells, including BC cells [8–10]. In particular, GPER signaling may trigger transcriptional events toward the stimulation of growth, migration, invasion and pro-inflammatory responses in BC cells [10–15]. Of note, GPER has been implicated in both the resistance to the ER antagonist tamoxifen and the up-regulation of aromatase levels, therefore leading to proliferative effects in tamoxifen-resistant BC cells [16–18]. In line with these findings, increased expression of GPER has been considered as a clinicopathological determinant of poor prognosis in patients with BC treated with endocrine therapy [19, 20].

The progression of cell cycle driven by estrogens mainly relies on the action of the cyclin D1-cyclin dependent kinase (CDK) 4/6 [21]. Accordingly, the CDK4/6 inhibitors named palbociclib, ribociclib and abemaciclib have been approved by the Food and Drug Administration (FDA) and the European Medicines Agency (EMA) for the management of ER-positive and HER2-negative advanced BC in combination with endocrine therapy [22]. In particular, palbociclib received an accelerated approval for the treatment of postmenopausal women exhibiting ER-positive and HER2-negative advanced BC on the basis of the results obtained by PALOMA-1 and PALOMA-2 clinical trials [23, 24]. Both studies reported a significant increase of the progression-free survival in patients treated with the aromatase inhibitor (AI) letrozole in combination with palbociclib respect to AI alone [25, 26]. The ensuing PALOMA-3 trial has also indicated that palbociclib in addition to the ER inhibitor fulvestrant improves overall survival (OS) in ER-positive and HER2-negative BC patients showing disease progression after endocrine therapy [27]. Nonetheless, resistance to CDK4/6 inhibitors, used either alone or in various combination regimens, appears to be almost inevitable [28, 29]. For instance, three phase III randomized studies evaluated the addition of CDK4/6 inhibitors to adjuvant endocrine therapy in patients eligible for high-risk adjuvant hormone therapy to prevent recurrence [30–32]. Of these, two studies (PALLAS and PENELOPE-B) assessed the combination of palbociclib with endocrine therapy, while one (MonarchE) evaluated the addition of abemaciclib to endocrine therapy. The duration of adjuvant therapy with CDK4/6 inhibitors was 2 years in the PALLAS and MonarchE studies, and 1 year in the PENELOPE-B study [30–32]. Among these three studies, only MonarchE demonstrated a statistically significant benefit in terms of invasive disease-free survival and distant disease-free survival from the addition of abemaciclib for 2 years in combination with hormone therapy compared to hormone therapy alone [32].

To date, diverse mechanisms have been involved in the resistance to palbociclib, including the overexpression of CDK4 as well as cyclins D1 and E, the loss of Retinoblastoma protein (Rb) activity, the aberrant up-regulation/activation of the Fibroblast Growth Factor Receptor (FGFR) and the Epidermal Growth Factor Receptor (EGFR), the mutations of ERα (ESR1) and Phosphatidylinositol-4,5-Bisphosphate 3-Kinase Catalytic Subunit Alpha (PIK3CA) [33–37]. Overall, a comprehensive understanding of the molecular events implicated in the resistance of BC to palbociclib is still lacking.

It is well acknowledged that the functional liaison between BC cells and the components of the tumor microenvironment (TME) plays a pivotal role in cancer growth, progression and resistance to pharmacological interventions [38–40]. In this regard, previous pre-clinical and clinical studies have shown that the combination of tumor and TME-targeted therapies may significantly reduce disease progression, dissemination and drug resistance [41]. Cancer-associated fibroblasts (CAFs), which represent the most abundant cell population within the breast TME, may serve as a source of hormones, growth factors, inflammatory molecules and other mediators of paracrine stimulatory actions on tumor cells [42–46]. Indeed, CAFs contribute to the progression of BC as well as to the acquired resistance to various pharmacological interventions [38, 47, 48]. Therefore, therapeutic strategies targeting CAFs or hampering CAFs-secreted molecules have been proposed to rescue drug sensitivity of BC cells [49]. Yet, previous reports have indicated that fibroblasts may alter the sensitivity of BC cells to chemotherapeutics such as palbociclib, although without disclosing the mechanisms involved [50–52].

In the present study, we show that palbociclib-resistant BC cells are characterized, among diverse molecular features, by an increased expression of GPER, which is required for cell resistance to palbociclib. Co-culturing BC cells and CAFs derived from breast tumor specimens, we also demonstrate that GPER is involved in the functional interaction occurring in the tumor microenvironment toward the resistance to palbociclib treatment. Hence, our data suggest that GPER may be included among the players contributing to the intricate molecular events leading to palbociclib resistance in both BC cells and the surrounding microenvironment.

## Methods

### Cell cultures

MCF7 and T47D BC cells were provided by ATCC (Manassas, VA, USA), used less than 6 months after resuscitation, routinely tested and authenticated according to ATCC suggestions. MCF7 cells were maintained in DMEM/F12 (Dulbecco’s modified Eagle’s medium) with phenol red, supplemented with 5% fetal bovine serum (FBS) and 1% penicillin/streptomycin (Thermo Fisher Scientific, Monza, Italy). T47D cells were maintained in RPMI 1640 with phenol red supplemented with 5% FBS, 0.2 units/mL bovine insulin (Merck, Milan, Italy) and 1% penicillin/streptomycin (Thermo Fisher Scientific, Monza, Italy). Palbociclib-resistant MCF7 (MCF7/PalbR) and T47D (T47D/PalbR) cells were established by long-term culture in the continuous presence of palbociclib. Cells were subcultured every 1–2 weeks with 10% increments in drug concentration (from 100 nM to 1 μM). Resistant cells were established after 6 months and routinely maintained in the presence of 1 μM palbociclib.

CAFs were isolated, cultured and characterized from 10 invasive mammary ductal carcinomas and pooled for the subsequent studies, as previously described [53]. Briefly, specimens were cut into 1-2 mm diameter pieces, placed in a digestion solution (400 IU collagenase, 100 IU hyaluronidase, 10% FBS, antibiotics and antimycotics) (Thermo Fisher Scientific, Monza, Italy) and incubated overnight at 37 °C. Cells were then separated by differential centrifugation at 90×g for 2 min. The supernatant containing fibroblasts was centrifuged at 485×g for 8 min, the pellet obtained was suspended in DMEM/F12 with phenol red, supplemented with 10% FBS and 1% penicillin/streptomycin (Thermo Fisher Scientific, Monza, Italy) and cultured at 37 °C and 5% CO_2_. CAFs were then expanded into 10-cm Petri dishes and stored as cells passaged for three population doublings within a total of 7 to 10 days after tissue dissociation. Primary cell cultures of fibroblasts were characterized by immunofluorescence with human anti-vimentin (V9; 1:500) and human anti-cytokeratin 14 (LL001) (Santa Cruz Biotechnology, DBA, Milan, Italy; 1:250). FAPα antibody (H-56, Santa Cruz Biotechnology, DBA, Milan, Italy; 1:500) was used to assess fibroblast activation (data not shown). We used CAFs passaged for up to 10 population doublings for the experiments, to minimize clonal selection and culture stress, which could occur during extended tissue culture. All cell lines were grown in a 37 °C incubator with 5% CO_2_.

### Reagents

Palbociclib (PD-0332991) and gefitinib were purchased from Merck (Milan, Italy), the MEK inhibitor trametinib was obtained from MedChemExpress (DBA, Milan, Italy), the CellTracker™ dyes CM-DiI and Green CMFDA were purchased from Thermo Fisher Scientific (Monza, Italy). All compounds were dissolved in dimethyl sulfoxide (DMSO).

### Plasmids and gene silencing experiments

For gene silencing experiments, cells were transfected for 36 h with control shRNA or specific shRNA sequence for each target gene using TurboFect™ Transfection Reagent (Thermo Fisher Scientific, Monza, Italy) according to the manufacturer’s instructions. For the generation of cell lines stably silenced for EGFR (MCF7/PalbR/shEGFR and T47D/PalbR/shEGFR) or GPER expression (MCF7/PalbR/shGPER and T47D/PalbR/shGPER) and a matched cell line harboring the control shRNA (MCF7/PalbR/shRNA and T47D/PalbR/shRNA), cells were transfected as described above and then treated with 1 μg/mL puromycin (Merck, Milan, Italy) in order to select stably-silenced cell clones. Thereafter, these cell lines were cultured in the presence of 250 ng/mL puromycin to avoid loss of plasmids. The SureSilencing™ shRNA plasmids for EGFR and the respective control plasmid (shRNA) were purchased from Superarray Bioscience Corporation (Frederick, MD, USA). The shRNA plasmids for GPER (TRCN0000235159, target sequence: TCTCGTGCCTCTACACCATCT; TRCN0000235161 target sequence: ATGAGCTTCGACCGCTACATC) and the respective control plasmid (shRNA, pLKO.1-puro non-target shRNA) were purchased from Merck (Milan, Italy).

### Gene expression studies

Total RNA was extracted, and cDNA was synthesized by reverse transcription, as previously described [54]. The expression of selected genes was quantified by real-time PCR using platform Quant Studio7 Flex Real-Time PCR System (Thermo Fisher Scientific, Monza, Italy). Gene-specific primers were designed using Primer Express version 2.0 software (Applied Biosystems) and are as follows: 5’-AGAGGGCATGGTGGAGATCTT-3’ (ESR1 forward) and 5’-CAAACTCCTCTCCCTGCAGATT-3’ (ESR1 reverse); 5′- ACACACCTGGGTGGACACAA-3′ (GPER forward) and 5′- GGAGCCAGAAGCCACATCTG -3′ (GPER reverse); 5′-TCCGTGAGTTGATCATCGAATT-3′ (EGFR forward) and 5′-GCATTCTTTCATCCCCCTGAA -3′ (EGFR reverse); 5′-AAGCCACCCCACTTCTCTCTAA-3′ (ACTB forward) and 5′-CACCTCCCCTGTGTGGACTT-3′ (ACTB reverse). Assays were performed in triplicate and the results were normalized for actin beta (ACTB) expression and then calculated as fold induction of RNA expression. PCR arrays were performed using a TaqMan™Human Chemokines Array (Thermo Fisher Scientific, Monza, Italy) according to the manufacturer’s instructions. The amplification reactions were carried out using platform Quant Studio7 Flex Real-Time PCR System (Thermo Fisher Scientific, Monza, Italy) and results were then analyzed on DataAssist software.

### Western blot analysis

Cells were grown in 10-cm dishes and then lysed as previously described [55]. Equal amounts of whole-protein extract were resolved on an 8% or 10% SDS-polyacrylamide gel and transferred to nitrocellulose membranes (Merck, Milan, Italy), which were probed with primary antibodies against ERα (F-10), EGFR (A-10), c-Fos (E-8), EGR1 (S-25), Cyr61 (A-10), p21 (H164), phosphorylated ERK1/2 (E-4), ERK2 (C-14), and β-actin (AC-15) (Santa Cruz Biotechnology, DBA, Milan, Italy), GPER (AB137479; Abcam, DBA, Milan, Italy), pAKT (4060) and AKT (9272) (Cell Signaling, Euroclone, Milan, Italy), cyclin D1 (TA801655) and cyclin E1 (AP06082PU-N) (purchased from OriGene Technologies, DBA, Milan, Italy) , and then revealed using the chemiluminescent substrate for western blotting Clarity™ Western ECL Substrate (Bio-Rad, Milan, Italy). For cytosolic and nuclear extracts, cells were lysed using 300 μl of cytosolic buffer (50 mM HEPES pH 7.5, 150 mM NaCl, 1% Triton X-100, 1.5 mM MgCl2, 1 mM EGTA, pH 7.5, 10% glycerol) with protease inhibitors (1.7 mg/ml aprotinin, 1 mg/ml leupeptin, 200 mmol/liter phenylmethylsulfonyl fluoride, 200 mmol/liter sodium orthovanadate and 100 mmol/liter sodium fluoride). Following centrifugation (14,000 g, 4 °C, 10 min), the supernatant was referred to as cytoplasmic fraction and the pellet containing nuclei was resuspended in high salt buffer (20 mM HEPES pH 7.9, 25% [v:v] glycerol, 420 mM NaCl, 1.5 mM MgCl2, 0.2 mM EDTA and protease inhibitors). For the extraction of nuclear proteins, the obtained solution was vortexed thoroughly, incubated overnight with agitation and centrifugated at 14000 g, 4 °C for 10 min. Equal amounts of the collected super-natant, which represent the nuclear fraction, were then run on 10% SDS-PAGE and western blot analysis was performed as described above. The purity of the nuclear fraction was confirmed by immunoblotting with primary antibodies against β-actin (AC-15; 1:4000) and anti-LMNB/Lamin (M-20; 1:2000) (Santa Cruz Biotechnol-ogy, DBA, Milan, Italy).

### Chromatin Immunoprecipitation (ChIP) assay

Cells were grown in 10-cm dishes, then cross-linked with 1% formaldehyde and sonicated. Supernatants were immuno-cleared with salmon DNA/protein A-agarose (Santa Cruz Biotechnology, DBA, Milan, Italy) and immunoprecipitated with nonspecific IgG or anti-EGFR (A-10; Santa Cruz Biotechnology, DBA, Milan, Italy). Pellets were washed, eluted with a buffer consisting of 1% SDS and 0.1 mol/L NaHCO3, and digested with proteinase K. DNA was obtained by phenol/chloroform extractions and precipitated with ethanol. The yield of target region DNA in each sample after ChIP was analyzed by real-time PCR. The primers used to amplify a region containing an AT-rich DNA consensus sequence located into the GPER promoter were: 5′-GCCAGGCTCACTTCAAGGAGA-3′ (Fw) and 5′-GTCTCTGCACCGTGCAGCTTT-3′ (Rv). Data were normalized to the input for the immunoprecipitation and the results were reported as fold changes respect to nonspecific IgG.

### Cell cycle analysis

To analyze cell cycle distribution, cells (1 × 10^5^) were cultured in medium containing 2.5% charcoal-stripped FBS in 6-well plates and then exposed to treatments, as indicated. Thereafter, cells were pelleted, washed with PBS, fixed in 50% methanol overnight at -20 °C, and then stained with a solution containing 50 μg/mL propidium iodide (PI) in 1×PBS, 20 U/mL RNAse-A and 0.1% Triton (Merck, Milan, Italy). Cell cycle phases were estimated as a percentage on a total of 10000 events. Samples were then analyzed with CytoFLEX flow cytometry (Beckman-Coulter, Milan, Italy).

### Proliferation assay

Cells (4 × 10^4^) were seeded in 24-well plates in regular growth medium and, once they had attached, were exposed to treatments, as indicated. Treatments were renewed every day and the proliferation rate was calculated counting the cells on day 4 by using the Countess Automated Cell Counter, as recommended by the manufacturer’s protocol (Thermo Fisher Scientific, Monza, Italy). For 2D cell co-cultures, immediately before seeding, MCF7 cells and CAFs were stained with CM-DiI and Green CMFDA CellTracker™ dyes, respectively, according to the manufacturer’s instructions (Thermo Fisher Scientific, Monza, Italy). Treatments were renewed every day, and transfections were renewed after 36 h, when required. The proliferation rate of MCF7 cells was calculated counting the cells on day 4 by using the Cytation 3 Cell Imaging Multimode reader (BioTek, AHSI, Milan Italy).

### Spheroid formation assay

For MCF7 and T47D spheroid generation, 100 μL/well of cell suspensions (1x10^4^ cells/well) were dispensed into 2% agar coated 96-well plates. Three days after seeding, tumor spheroids (a single spheroid per well) were exposed to treatments, when required, and a 50% medium and treatment replenishment was performed every 2 days. Images were obtained on day 7 using a conventional inverted microscope, thereafter cell number per spheroid was determined by trypsinizing 6 different spheroids, mixing the cell suspension with trypan blue and counting the number of viable cells by using the Countess Automated Cell Counter (Thermo Fisher Scientific, Monza, Italy). The total number of cells obtained was divided by the number of trypsinized spheroids. Co-culture spheroids were obtained by simultaneously seeding MCF7 or T47D cells and CAFs (previously transfected for 36 h with control shRNA or shGPER) into 2% agar coated 96-well plates (1.5x10^4^ cells/well, 1:2 ratio). A 50% medium and treatment replenishment was performed every 24 h. In order to distinguish the two cell lines, immediately before seeding BC cells and CAFs were stained with CM-DiI and Green CMFDA CellTracker™ dyes, respectively, according to the manufacturer’s instructions (Thermo Fisher Scientific, Monza, Italy). Fluorescent images of spheroids were captured and analyzed using the Cytation 3 Cell Imaging Multimode reader (BioTek, AHSI, Milan Italy) on day 4.

### Colony formation assay

Cells (3 × 10^3^) were seeded in 6-well plates in medium containing 5% charcoal-stripped FBS and then exposed to treatments, as indicated. Treatments were renewed every 2 days. After 10 days, cells were washed with PBS, fixed in acetone:methanol (1:1) for 3 min at room temperature and then stained with 0.1% Crystal Violet (Merck, Milan, Italy) for 20 min. A total of 10 pictures for each condition was detected by using a digital camera and the colony number was measured by the ImageJ program.

### Molecular docking

Simulations of the binding of palbociclib, G-1 and estradiol (E2) were carried out on a structural model of GPER [11] built by using GPCR-I-TASSER, which is an algorithm specifically designed to model G protein-coupled receptors [56]. The molecular structure of the ligands was built by using the modeling software Avogadro [57]. Docking calculations were performed by using AutoDock Vina 1.1.2 [58]. Preliminary conversion of the structures from the PDB format was carried out by using the graphical interface AutoDock Tools 1.5.6 [59]. During the conversion, polar hydrogens were added for the enzyme, and apolar hydrogens of all compounds were merged to the carbon atom they were attached to. Full flexibility was guaranteed for the ligands, resulting in 5, 2 and 2 rotatable dihedral angles for palbociclib, G-1 and E2, respectively. To account for the binding in any possible region of GPER, a search volume including the whole protein (40 Å × 48 Å × 70 Å) was considered, with a grid spacing of 1 Å. In each case a single simulation run was carried out at very high exhaustiveness, 16 times larger than the default value [60]. The Molecular Graphics System PyMOL was used to visualize the protein structure and ligand binding (PyMOL Molecular Graphics System, Version 2.1.1, Schrödinger, LLC inc). Intermolecular interactions were evaluated by using the automated protein–ligand interaction profiler (PLIP) [61].

### Data sources

Investigations were performed using The Cancer Genome Atlas (TCGA) and Molecular Taxonomy of Breast Cancer International Consortium (METABRIC) datasets [62, 63]. Data was downloaded on the 22^th^ of December 2023. Patients data and gene expression data (RNA Seq V2 RSEM) of the Invasive Breast Cancer Cohort of the TCGA project were retrieved from UCSC Xena (https://xenabrowser.net/). The clinical information and the microarray gene expression data (Log_2_-transformed intensity values) of the METABRIC cohort (n. 2509) were downloaded from the cBioPortal for Cancer Genomics (http://www.cbioportal.org/). Samples of the TCGA cohort (n. 1247) were filtered by the “sample type” in order to obtain exclusively information on the tumor tissues (n. 1101). Subsequently, patients of both TCGA and METABRIC datasets were classified on the basis of the presence or absence of the ER detected by immunohistochemistry. Gene expression and clinical information were also filtered for missing values. Data on the stromal scores of the TCGA BC samples, calculated by the ESTIMATE algorithm, was downloaded from https://bioinformatics.mdanderson.org/estimate/. ER-positive BCs were classified into high- and low-score groups according to the median value of the stromal scores.

### Survival analysis

The survival analysis on ER-positive BC patients was assessed by using gene expression data, OS and relapse free survival (RFS) information of the METABRIC dataset. Samples were filtered for the vital status and patients classified as “died of other causes” were excluded. ER-positive BCs were divided into high and low expression groups according to the median expression values of both GPER and EGFR. The Kaplan-Meier survival plots were generated by using the survival and the survminer R packages, a log-rank test was used to determine differences between the survival curves. A value of *p* < 0.05 was considered statistically significant.

### K-means clustering

K-means clustering was performed on ER-positive BC samples of the METABRIC dataset by using the *kmeans()* function in R Studio. The min-max normalization was applied to gene expression data and the silhouette method was employed to generate the optimal clustering number (Additional File 1A). Thereafter, by applying the *kmeans()* function the clustering label of each sample was obtained. On the basis of the OS data, the Kaplan-Meier survival curves of the two clusters of patients were drawn and log-rank p-value was calculated. Values of *p* < 0.05 was considered statistically significant.

### Statistical analysis

The statistical analysis was performed by using ANOVA followed by Newman-Keuls’ test to determine differences in means. All bioinformatics analyses were carried out using R Studio (version 4.1.3). Box plots and stacked bar charts were performed with the tidyverse package (https://www.tidyverse.org/packages/) and the related statistical analysis was carried out by using the Wilcoxon and chi-squared tests. Heatmap was drawn with the pheatmap package in R Studio.

## Results

### Generation of palbociclib-resistant BC cells

**Fig. 1:**
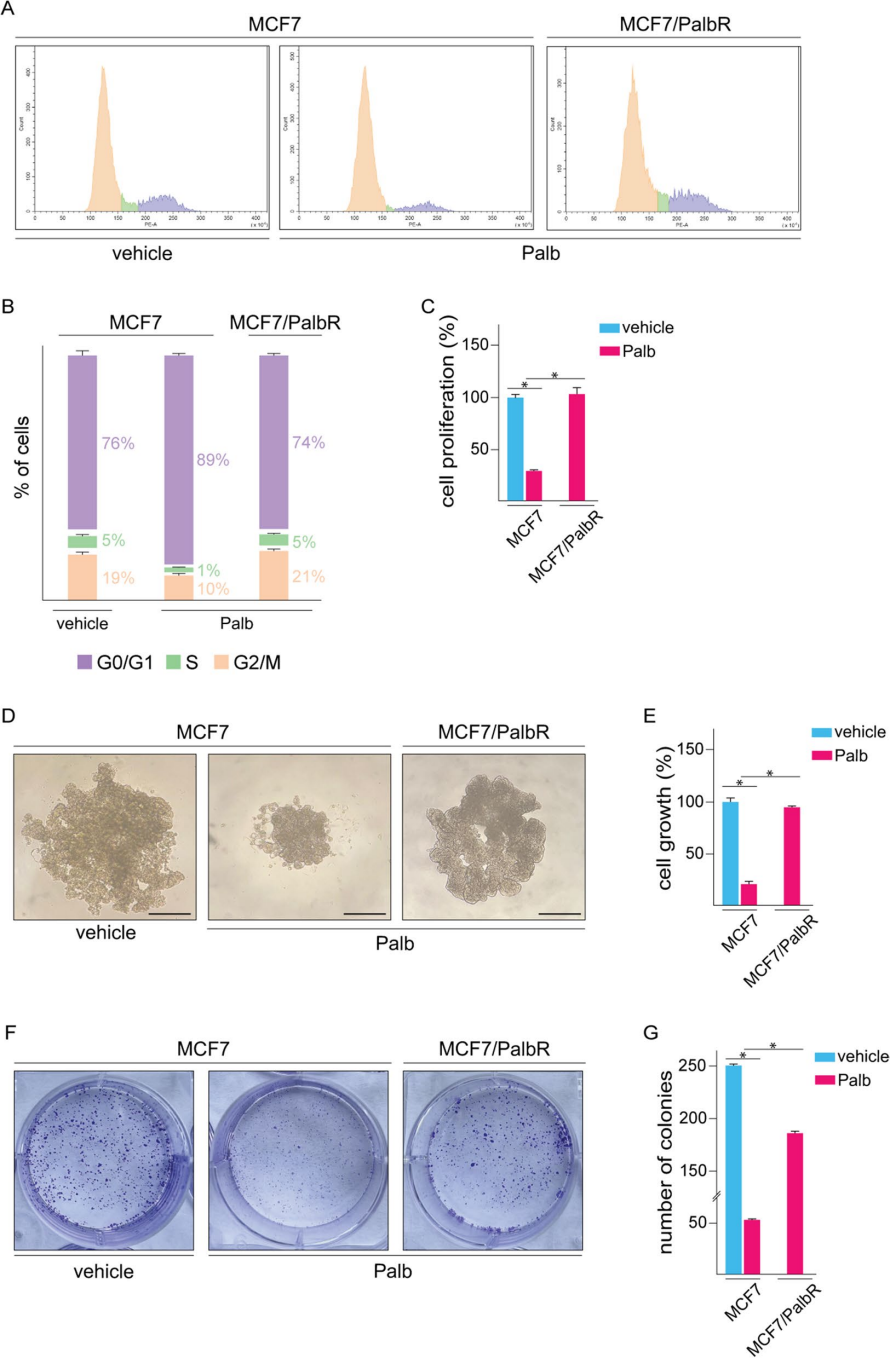
Establishment of palbociclib-resistant MCF7 (MCF7/PalbR) BC cells. **A** Cell cycle analysis performed by flow cytometry in MCF7 and MCF7/PalbR cells treated with vehicle or 1 μM palbociclib (Palb) for 12 h. **B** Percentage of cells in G0/G1, S and G2/M phases of cell cycle. **C** Proliferation of MCF7 and MCF7/PalbR cells after 3 days treatment with vehicle or 1 μM palbociclib (Palb). Values of vehicle-treated MCF7 cells were set as 100% upon which cell viability was determined. **D** Representative pictures of spheroids (a single spheroid/well) from the MCF7 and MCF7/PalbR spheroid cultures grown on agar-coated plates and exposed for 6 days to vehicle or 1 μM palbociclib (Palb), as indicated. Scale bar: 500 μm. **E** Quantification of spheroid growth. Values of vehicle-treated MCF7 cells were set as 100% upon which spheroid growth was determined. **F** Colony formation assay in MCF7 and MCF7/PalbR cells exposed to vehicle or 1 μM palbociclib (Palb). Plates were stained with Crystal Violet and colonies were counted following 10 days of incubation (**G**). Values represent the mean ± SD of three independent experiments performed in triplicate. (*) indicates *p* < 0.05

Cell cycle regulators are currently valuable targets of CDK4/6 inhibitors in ER-positive BC; nevertheless, the occurrence of resistance remains a prevalent challenge [64]. In order to gain novel insights on the molecular events involved in BC resistance to the largely used CDK4/6 inhibitor palbociclib, we began the present study by establishing two palbociclib-resistant ER-positive BC cell lines. In this vein, MCF7 and T47D BC cells were exposed to increasing doses of palbociclib (from 100 nM to 1 μM). Upon approximately 6 months of treatment, Palbociclib-resistant MCF7 (MCF7/PalbR) and T47D (T47D/PalbR) cells were established. Performing flow cytometric analysis of PI-staining, we observed that palbociclib induces the arrest of MCF7 cell cycle in the G1 phase, whereas in MCF7/PalbR cells no change in cell cycle distribution was noticed (Fig. 1A-B). Accordingly, palbociclib treatment had no effect on the proliferation (Fig. 1C), spheroid expansion (Fig. 1D-E) and colony-forming ability (Fig. 1F-G) of MCF7/PalbR cells, as opposed to what is assessed in MCF7 cells (Fig. 1C-G). The establishment of palbociclib resistance in T47D/PalbR cells was also ascertained (Additional File 2A-B).

### Palbociclib-resistant BC cells display increased EGFR and GPER expression

**Fig. 2:**
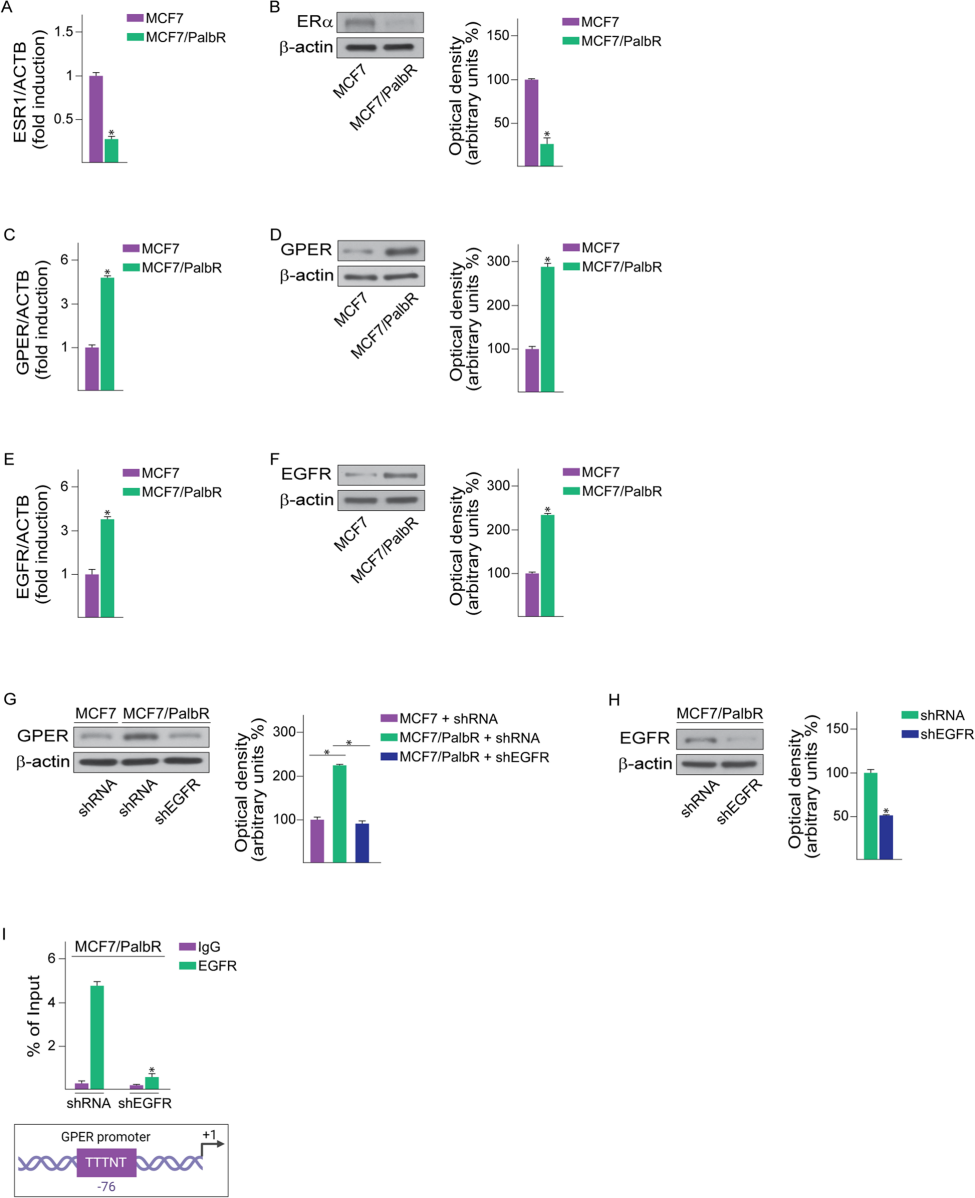
EGFR mediates the increase of GPER expression in MCF7/PalbR cells. mRNA (**A**) and protein (**B**) expression of ERα in MCF7 and MCF7/PalbR cells, as evaluated by real-time PCR and immunoblotting assays, respectively. mRNA (**C**, **E**) and protein (**D**, **F**) levels of GPER and EGFR in MCF7 and MCF7/PalbR cells, as evaluated by real-time PCR and immunoblotting, respectively. In RNA experiments, values are normalized to the actin beta (ACTB) expression and shown as fold changes of mRNA expression in MCF7/PalbR respect to MCF7 cells. **G** GPER protein expression in MCF7 and MCF7/PalbR cells transiently transfected with a control shRNA or a shEGFR plasmid, as indicated. **H** Efficacy of EGFR silencing in MCF7/PalbR cells. Side panels show densitometric analyses of the blots normalized to β-actin, which served as loading control. **I** Recruitment of EGFR to the AT-rich sequence located within the GPER promoter, as ascertained by ChIP assay in MCF7/PalbR cells transiently transfected with a control shRNA or a shEGFR plasmid. In control samples, nonspecific IgGs were used instead of the primary antibody. The amplified sequences were evaluated by real-time PCR. Values represent the mean ± SD of three independent experiments performed in triplicate. (*) indicates *p* < 0.05. Created with BioRender.com

In order to assess the peculiar features of palbociclib-resistant BC cells, we first ascertained that ERα mRNA and protein levels are down-regulated in both MCF7/PalbR (Fig. 2A-B) and T47D/PalbR (Additional File 2C) cells respect to their parental counterparts, in accordance with previous studies [65–67]. Considering that the estrogen receptor GPER mediates estrogenic signaling in different cell contexts [10, 15, 68], we aimed to evaluate whether the expression of GPER may be altered in palbociclib-resistant BC cells. Reminiscing previous RNA-sequencing data [69], we found that the expression of GPER is increased at both the mRNA and protein levels in MCF7/PalbR (Fig. 2C-D) and T47D/PalbR cells (Additional File 2D) respect to the parental MCF7 and T47D cells. Moreover, the levels of GPER augmented in both the cytoplasmic and nuclear compartment of MCF7/PalbR (Additional File 3) and T47D/PalbR cells (data not shown) respect to their parental counterparts, as demonstrated by subcellular fractionation studies. A multifaceted interplay between GPER and EGFR has been demonstrated in diverse cancer cell lines, including BC cells [70–72]. Moreover, the up-regulation of EGFR has been previously established in BC cells resistant to palbociclib [36], thus we hypothesized the involvement of EGFR in the regulation of GPER. In this regard, we first ascertained that an increased expression of EGFR occurs in MCF7/PalbR (Fig. 2E-F) and T47D/PalbR (Additional File 2D) cells respect to their palbociclib-sensitive counterparts. Thereafter, we found that the silencing of EGFR expression leads to lowered GPER levels in both MCF7/PalbR (Fig. 2G-H) and T47D/PalbR cells (Additional File 2E). In accordance with these findings and previous studies demonstrating that the activation of diverse pro-survival transduction pathways has been associated to CDK4/6 inhibitor resistance [73, 74], we observed an increased phosphorylation of certain EGFR downstream signaling proteins [75], such as extracellular-related kinase (ERK) and phosphoinositide 3-kinase (PI3K)/Akt (data not shown). Considering that EGFR may also act as transcription factor [71, 76, 77], we ascertained by ChIP assays the recruitment of EGFR to an AT-rich consensus sequence located within the promoter of GPER in MCF7/PalbR cells (Fig. 2I). Further corroborating the aforementioned findings, the recruitment of EGFR to the promoter of GPER was no longer evident upon knocking down the expression of EGFR (Fig. 2I).

**Fig. 3:**
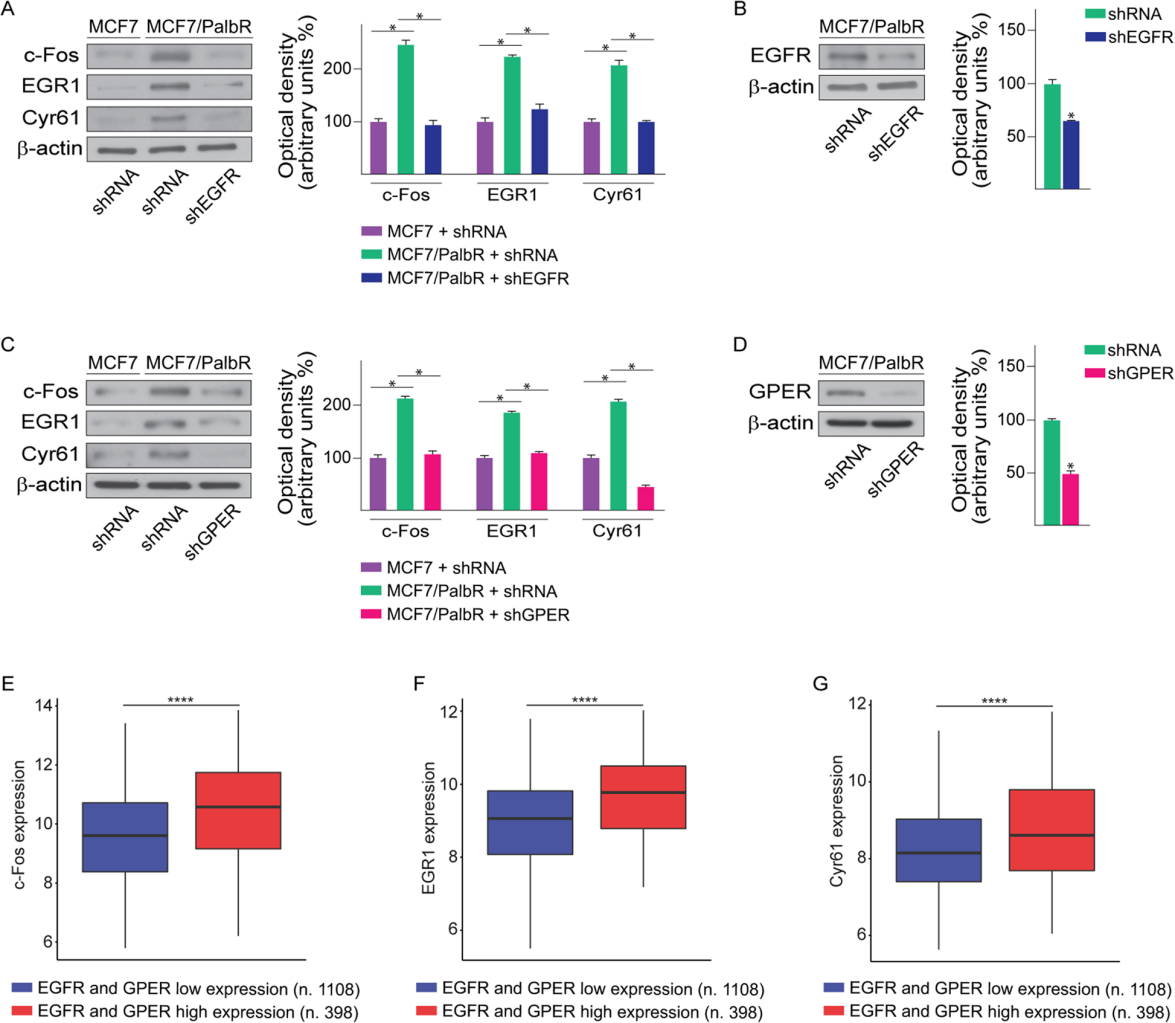
The up-regulation of c-Fos, EGR1 and Cyr61 in MCF7/PalbR cells relies on both EGFR and GPER. **A** Immunoblots of c-Fos, EGR1 and Cyr61 in MCF7 and MCF7/PalbR cells transiently transfected with a control shRNA or a shEGFR plasmid, as indicated. **B** Efficacy of EGFR silencing in MCF7/PalbR cells. **C** Protein levels of c-Fos, EGR1 and Cyr61 in MCF7 and MCF7/PalbR cells transiently transfected with a control shRNA or a shGPER plasmid, as indicated. **D** Efficacy of GPER silencing in MCF7/PalbR cells. Side panels show densitometric analyses of the blots normalized to β-actin, which served as loading control. c-Fos (**E**), EGR1 (**F**) and Cyr61 (**G**) mRNA levels in METABRIC ER-positive BC patients with elevated expression of both EGFR and GPER (median values were used as threshold). (*) indicates *p* < 0.05. (****) indicates *p* < 0.0001

**Fig. 4:**
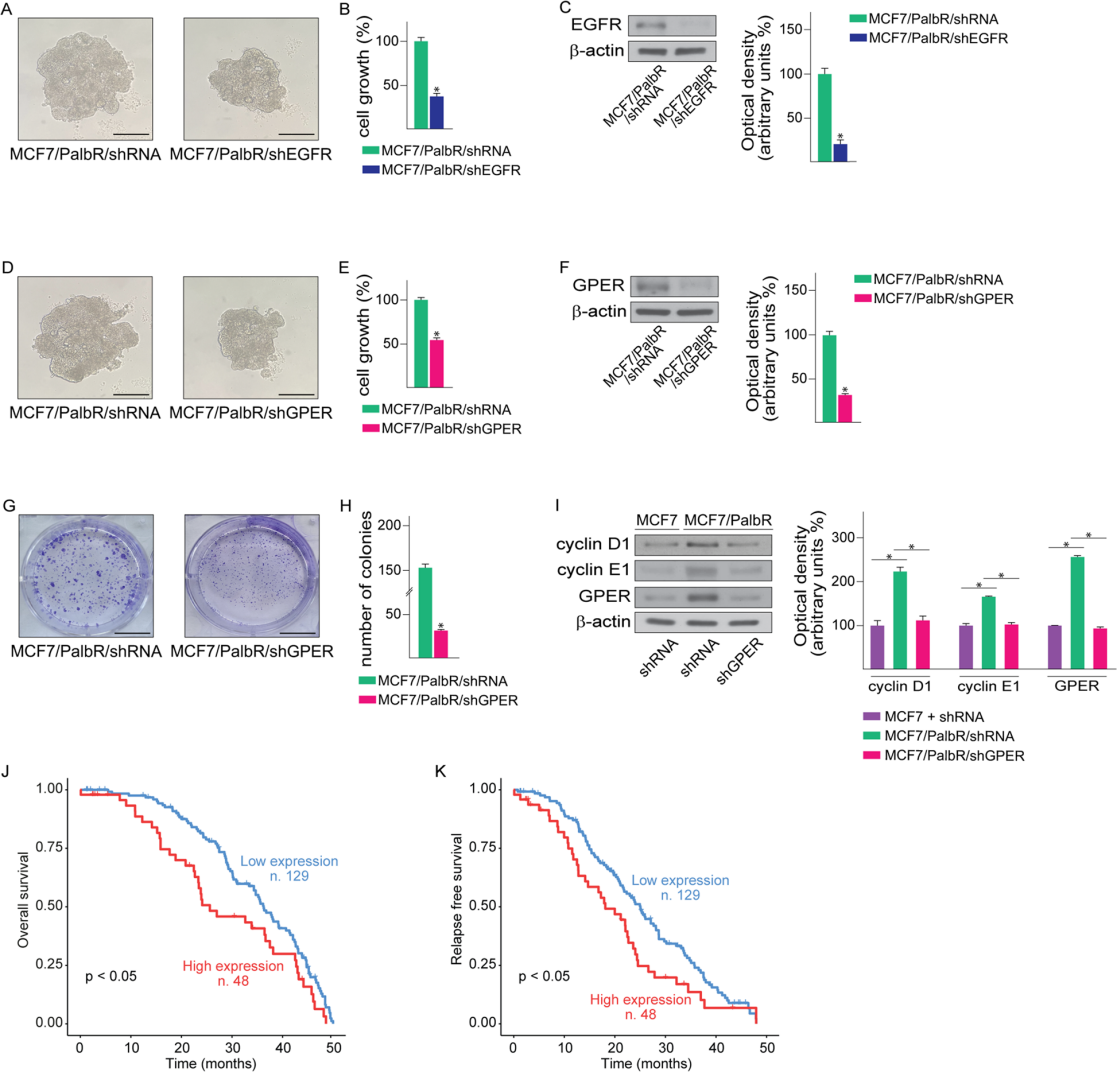
EGFR or GPER silencing restore palbociclib sensitivity in MCF7/PalbR cells. **A** Representative pictures of spheroids (a single spheroid/well) from MCF7/PalbR/shRNA and MCF7/PalbR/shEGFR spheroid cultures grown for 6 days on agar-coated plates. **B** Quantification of spheroid growth; values of MCF7/PalbR/shRNA cells were set as 100% upon which the number of MCF7/PalbR/shEGFR cells was determined. **C** Efficacy of EGFR silencing in MCF7/PalbR/shEGFR cells. **D** Representative pictures of spheroids (a single spheroid/well) from the MCF7/PalbR/shRNA and MCF7/PalbR/shGPER spheroid cultures grown for 6 days on agar-coated plates. Scale bar 500 μm. **E** Quantification of spheroid growth; values of MCF7/PalbR/shRNA cells were set as 100% upon which the number of MCF7/PalbR/shGPER cells was determined. **F** Efficacy of GPER silencing in MCF7/PalbR/shGPER cells. **G** Colony formation assay in in MCF7/PalbR/shRNA and MCF7/PalbR/shGPER cells. Plates were stained with Crystal Violet and colonies were counted following 10 days of incubation. (**H**). **I** Protein levels of cyclin D1, cyclin E1 and GPER in MCF7/PalbR/shRNA and MCF7/PalbR/shGPER cells. Side panels show densitometric analyses of the blots normalized to β-actin, which served as loading control. Values represent the mean ± SD of three independent experiments performed in triplicate. (*) indicates *p* < 0.05. Kaplan-Meier survival curves representing the overall survival (**J**) and relapse-free survival (**K**) in ER-positive BC patients of the METABRIC database, based on low *vs* high EGFR and GPER mRNA levels (median values were used as threshold)

Altogether, these results suggest that EGFR is involved in the transcriptional regulation of GPER in palbociclib-resistant BC cells. In order to further corroborate the aforementioned results, we also evaluated the expression of certain GPER-target genes [78] in palbociclib-resistant BC cells. Immunoblotting assays showed that the expression of c-Fos, EGR1 and CYR61 is higher in MCF7/PalbR respect to MCF7 parental cells (Fig. 3A, C). Of note, the up-regulation of the aforementioned proteins was no longer evident upon silencing EGFR (Fig. 3A-B) or GPER (Fig. 3C-D) in MCF7/PalbR cells. Nicely fitting with these data, *in silico* analyses revealed that the expression of c-Fos, EGR1 and CYR61 is significantly higher in ER-positive BC patients displaying EGFR and GPER levels above the median value, compared to patients exhibiting the expression of both receptors below the median value (Fig. 3E-G). Next, we sought at evaluating whether EGFR and GPER may be implicated in the growth of palbociclib-resistant BC cells. To this aim, the expression of EGFR (Fig. 4A-C; Additional File 4A-C) or GPER (Fig. 4D-F; Additional File 4D-F) was stably silenced in MCF7/PalbR and T47D/PalbR cells. Remarkably, after knocking down these receptors, the spheroid expansion of MCF7/PalbR (Fig. 4A-F) and T47D/PalbR (Additional File4A-F) cells was impaired respect to cells stably transfected with a control shRNA construct. The aforementioned findings were also achieved by using a second shRNA targeting a different GPER sequence (data not shown). Similar results were observed in MCF7/PalbR and T47D/PalbR cells upon exposure to the EGFR inhibitor gefitinib (data not shown), in accordance with previous studies indicating that EGFR inhibition may blunt the proliferation of palbociclib resistant breast cancer cells [79]. GPER silencing was also able to weak the colony forming ability of palbociclib resistant cells (Fig. 4G-H; Additional File 4G-H). Accordingly, the protein levels of certain cell-cycle regulators, including cyclin D1 and cyclin E1, were down-regulated in GPER-silenced MCF7/PalbR (Fig. 4I) and T47D/PalbR (Additional File 4I). It is worth mentioning that even ER-positive BC patients displaying EGFR and GPER levels above the median value exhibit poor OS and RFS outcomes (Fig. 4J-K). These observations may corroborate our findings indicating that increased levels of both receptors, as observed in MCF7/PalbR and T47D/PalbR cells, may be involved in worse outcomes in breast cancer patients.

### Palbociclib triggers the ERK/c-Fos transduction pathway through GPER in CAFs

**Fig. 5:**
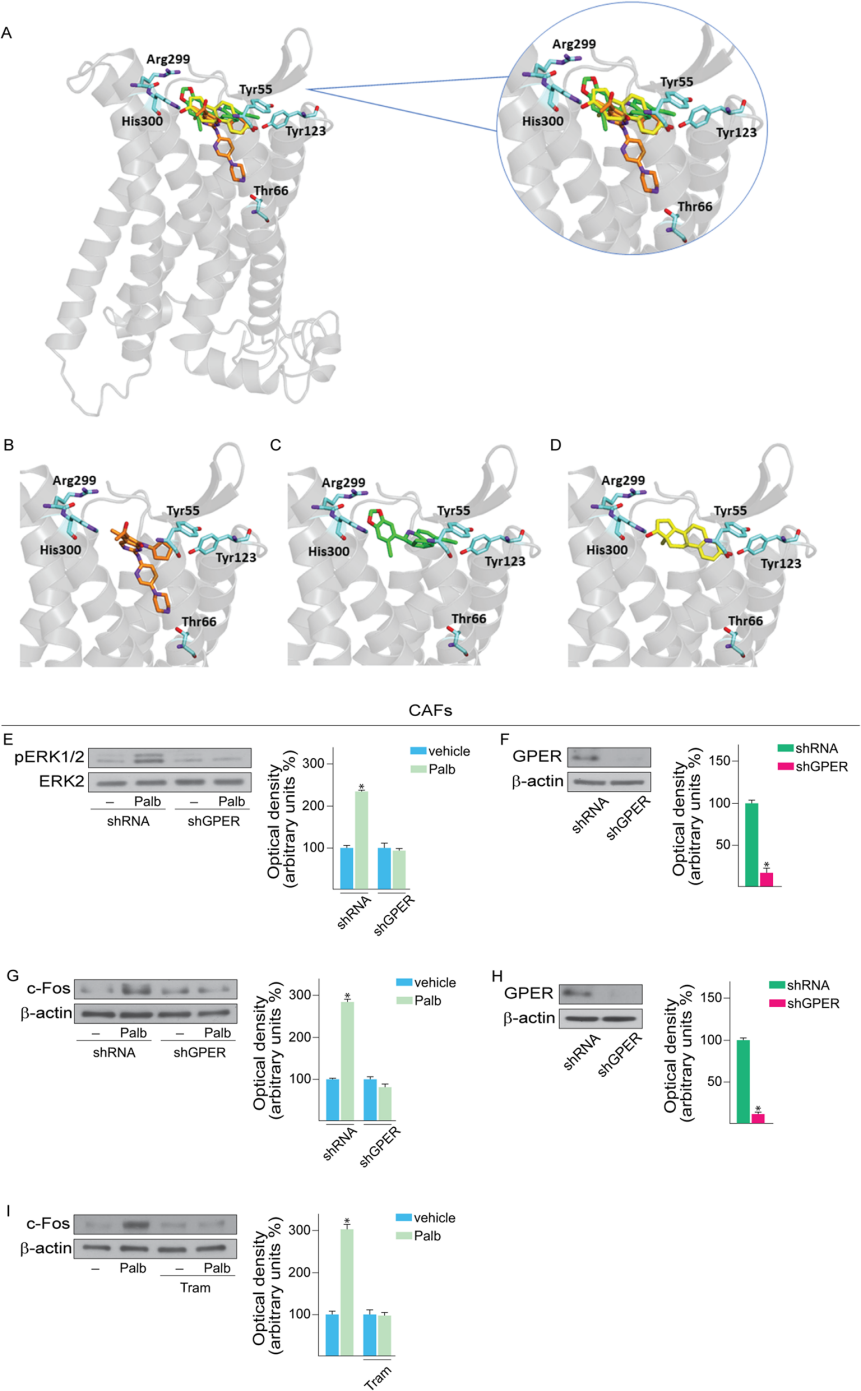
Palbociclib triggers the activation of GPER signaling in CAFs. **A** Superimposed binding modes of palbociclib (orange), G-1 (green) and E2 (yellow) in a GPER model, and details of the binding site. Protein backbone is represented as a ribbon and the key protein residues Tyr55, Thr66, Tyr123, Arg299 and His300 are in cyan. The ligands are also shown separately: palbociclib (**B**), G-1 (**C**) and E2 (**D**). **E** ERK1/2 phosphorylation in CAFs transiently transfected with a control shRNA or a shGPER plasmid and then exposed for 15 min to vehicle (–) or 1 μM palbociclib (Palb). (**F**, **H**) Efficacy of GPER silencing in CAFs. **G** c-Fos protein levels in CAFs transiently transfected with a control shRNA or a shGPER plasmid and thereafter exposed for 4 h to vehicle (–) or 1 μM palbociclib (Palb). **I** Immunoblot of c-Fos in CAFs treated with vehicle (-) or 1 μM palbociclib (Palb) in the presence or absence of 100 nM trametinib (Tram). Side panels show densitometric analyses of the blots normalized to ERK2 and β-actin that served as loading controls, as indicated. Values represent the mean ± SD of three independent experiments performed in triplicate. (*) indicates *p* < 0.05

In the context of biomolecular screening studies aimed at identifying novel GPER agonists and antagonists, we observed that palbociclib may interact with the binding site of GPER. In particular, we carried out molecular docking experiments on a 3D structural model of the protein, already used in our previous investigations in order to assess the binding ability of diverse GPER ligands [11, 80, 81]. The mode of interaction of palbociclib with GPER was compared to that of two known GPER agonists, G-1 and E2, which were also docked in the same receptor binding site. As shown in figure 5 (panels A-D), all compounds occupied the same region surrounded by protein residues Tyr55, Thr66, Tyr123, Arg299 and His300 within the protein cavity emerging from the transmembrane portion of the receptor. In particular, the cyclopentylpyridopyrimidinone system of palbociclib overlapped to both the tricyclic moiety of G-1 and the steroid skeleton of E2, with the appended cyclopentyl moiety binding to Tyr55 and Lys119 residues and the pyridopyrimidinone system binding to Gln54 and Pro303 through hydrophobic interactions. Furthermore, the pyrimidinepyrido portion of palbociclib penetrated deeper into the cavity of the GPER binding region, interacting through the distal nitrogen of piperazine with the Thr66 residue by a hydrogen bond. All of these interactions contribute to the stabilization of the ligand-receptor complex, as reflected in the binding energy value, which was even more favorable for palbociclib compared to known GPER ligands (-8.3, -7.6 and -7.4 kcal/mol for palbociclib, G-1 and E2 respectively; Table 1).

**Table 1.** Binding energy values and main interactions of palbociclib, G-1, and E2 with GPER.

	Structure	Binding energy (kcal/mol)	Interactions				
			Hydrogen Bonds				Hydrophobic Interactions
			GPER residues	Distance (Å)		Donor Angle (°)	GPER Residues
				H-A	D-A		
palbociclib		-8.3	Thr66	2.11	2.80	127.01	Gln54 Tyr55 Leu119 Pro303
G-1		-7.6	Tyr123 Arg299 His300	2.05 3.61 1.91	2.89 4.09 2.68	148.32 112.46 133.99	Tyr55 Tyr123 Phe206 Pro303
E2		-7.4	Tyr55 His300	3.21 2.15	3.98 3.01	137.44 144.78	Gln54 Tyr55 Pro303

Previous studies had ascertained that GPER signaling can be deemed as a mediator of stromal functions due to its ability to induce stimulatory effects in different cell components of the tumor microenvironment like CAFs [14, 49, 54, 82, 83]. On the basis of these results and other evidence showing that the interaction between BC and stromal cells may reduce the sensitivity to palbociclib [52], we sought to investigate whether palbociclib may act through GPER in breast CAFs. Reminiscing previous data showing that activated GPER triggers the ERK transduction signaling in CAFs [11, 84], we first assessed that the rapid ERK activation induced by palbociclib is prevented by silencing the expression of GPER (Fig. 5E-F). Thereafter, we determined that the up-regulation of well-known target genes of GPER like c-Fos (Fig. 5G-H), EGR1 and Cyr61 (Additional File 5A-B) by palbociclib is abrogated by silencing the expression of GPER .

In line with our previous findings demonstrating that the ERK transduction pathway regulates several GPER target genes including c-Fos [13, 84, 85], we also established that the MEK inhibitor trametinib prevents the up-regulation of c-Fos upon palbociclib treatment (Fig. 5I).

### The palbociclib-induced regulation of pro-inflammatory genes is mediated by GPER in CAFs and is involved in BC resistance

**Fig. 6:**
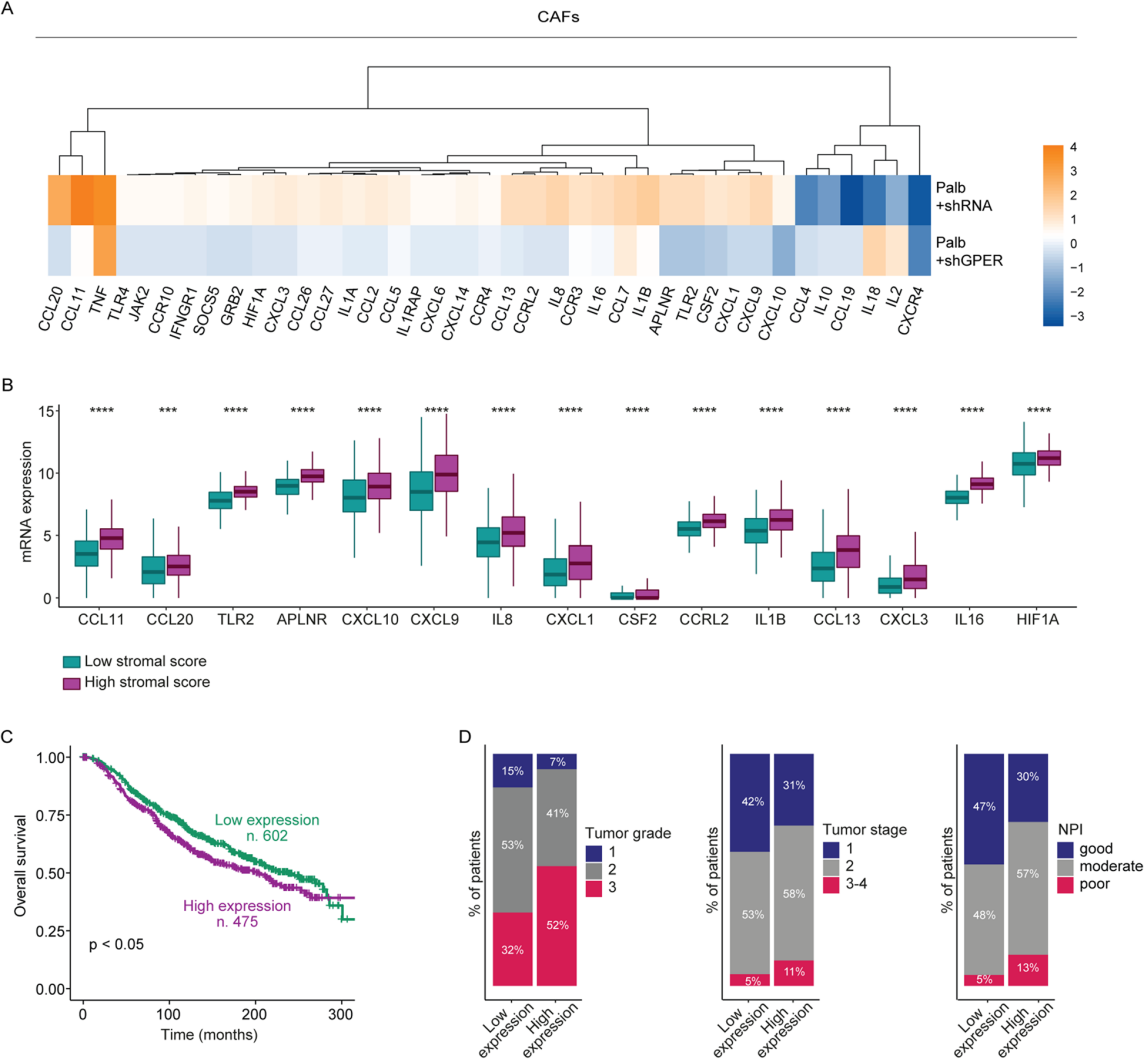
Palbociclib stimulates a pro-inflammatory gene expression profile through GPER in CAFs. **A** CAFs were transiently transfected with a control shRNA or a shGPER plasmid and then treated with vehicle or 1 μM palbociclib (Palb) for 8 h. Values were normalized to the 18S gene expression; the colors indicate the log_2_ fold changes of gene expression upon palbociclib respect to vehicle-treated cells, as indicated. **B** Multiple boxplot showing the differential expression of GPER-dependent pro-inflammatory genes in ER-positive BC samples of the TCGA dataset characterized by a low or high stromal score. **C** Kaplan Meier survival curves in METABRIC ER-positive BC patients exhibiting high expression levels of the GPER-regulated inflammatory genes, according to k-means clustering analysis. **D** ER-positive BC samples characterized by elevated expression of GPER-dependent pro-inflammatory genes display worse clinical features in terms of tumor grade, tumor stage and NPI (Nottingham Prognostic Index), as indicated. (***) indicates *p* < 0.001; (****) indicates *p* < 0.0001

CAFs secrete a variety of inflammatory cytokines, chemokines and growth factors as well as trigger the extracellular matrix (ECM) remodeling, thereby regulating key aspects of tumor biology like cancer cell proliferation, invasion, metastasis, angiogenesis and tumor-associated inflammation [44, 86–88]. Considering that GPER has been implicated in the regulation and release of inflammatory mediators as interleukin 1β (IL-1β) in breast CAFs [13, 14], we performed a TaqMan™Human Chemokines Array in order to provide insights on the inflammatory gene profile raised by palbociclib through GPER in CAFs. CAFs were transfected with a control shRNA or a shGPER plasmid and then exposed to palbociclib (Fig. 6A). A total of 15 genes showing at least 1.5-fold induction by palbociclib respect to vehicle in shRNA-transfected CAFs and a reduction of at least 50% in shGPER-transfected CAFs were observed (Table 2).

**Table 2:** GPER-regulated pro-inflammatory genes in CAFs

**Gene name**	**Palbociclib vs vehicle in shRNA-transfected CAFs** **(fold changes)**	**Palbociclib vs vehicle in** **shGPER-transfected CAFs** **(fold changes)**
CCL11	17.79	1.44
CCL20	7.24	0.82
TLR2	2.62	0.57
APLNR	2.46	0.59
CXCL10	1.64	0.41
CXCL9	2.77	0.73
IL8	2.96	0.90
CXCL1	2.40	0.74
CSF2	2.13	0.67
CCRL2	2.51	0.90
IL1B	3.63	1.36
CCL13	2.58	0.97
CXCL3	2.00	0.91
IL16	2.52	1.18
HIF1A	1.78	0.88

In order to assess whether the stromal milieu of ER-positive BC patients is enriched in the aforementioned genes, we performed bioinformatics analyses by using the TCGA cohort. Remarkably, we found that the 15 identified genes are up-regulated in ER-positive BC patients showing a high stromal infiltration (Fig. 6B). Thereafter, by k-means clustering we divided the population of ER-positive BC patients into two subgroups characterized by high or low expression of the abovementioned 15 inflammatory genes (Additional File 1B). Of note, patients belonging to the high gene expression cluster exhibited both poor prognosis (Fig. 6C) and worse clinical features in terms of tumor grade, tumor stage and Nottingham Prognostic Index (NPI) (Fig. 6D). Overall, these data suggest that the inflammatory genes triggered by palbociclib through GPER in CAFs might be taken into consideration as indicators of a poor clinical outcome in ER-positive BC patients.

**Fig. 7:**
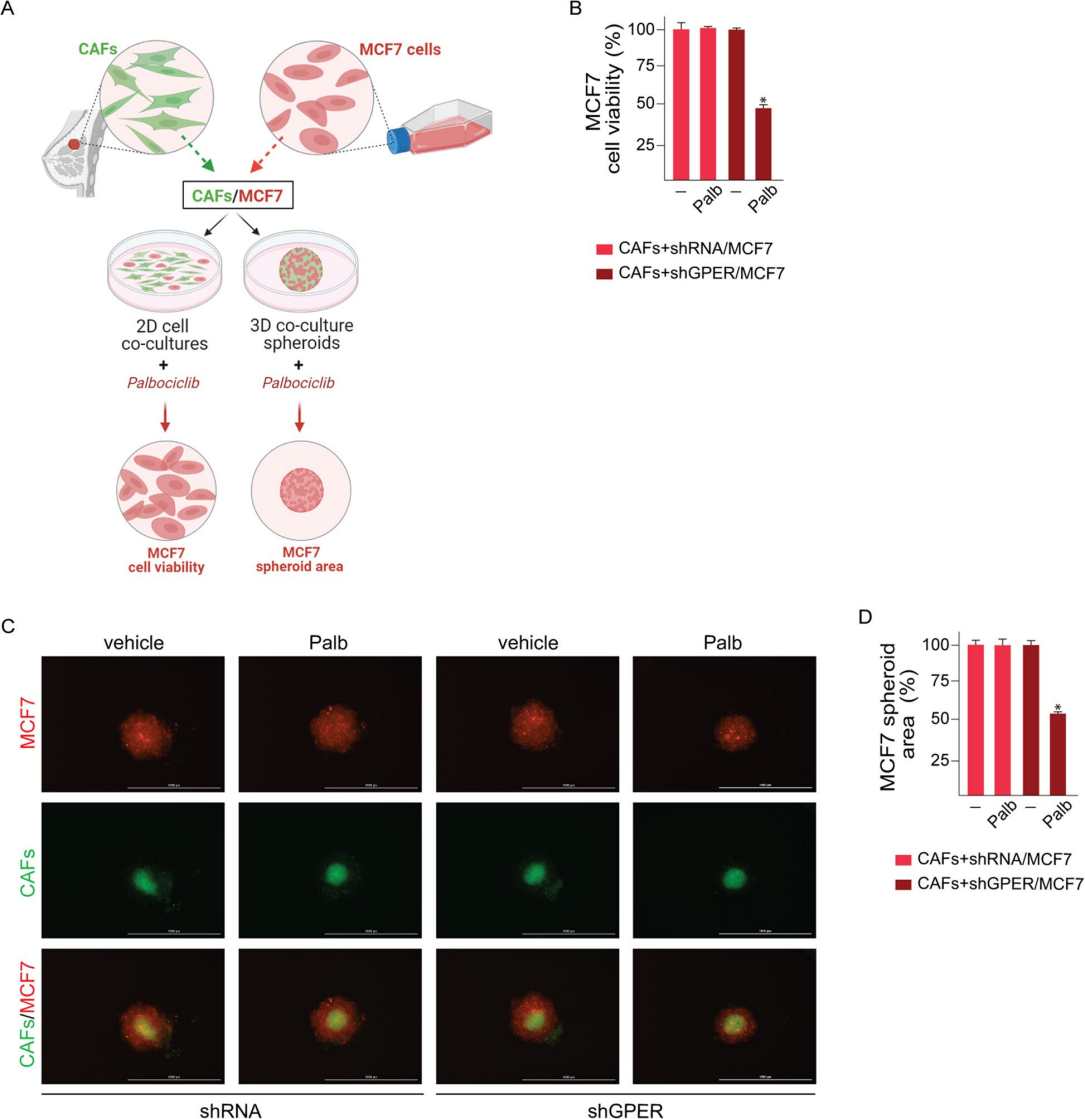
Palbociclib-treated BC cells acquire an increased survival capacity following GPER activation in CAFs. **A** Workflow of the 2D cell co-cultures and 3D co-culture spheroid assays. BC patient-derived CAFs, which were previously transfected with control shRNA or shGPER plasmids and stained with CellTracker™ Green CMFDA dye, were co-cultured with MCF7 cells previously stained with CellTracker™CM-DiI dye. Co-cultures were exposed for 3 days to vehicle or 1 μM palbociclib (Palb), then MCF7 cell number and spheroid areas were analyzed on day 4. Created with BioRender.com. **B** Viability of MCF7 cells after 3 days treatment with vehicle or 1 μM palbociclib (Palb) and 2D co-cultured with CAFs that were previously transfected with control shRNA or shGPER plasmids. Values of vehicle-treated MCF7 cells were set as 100% upon which cell viability was determined. **C** Representative pictures of MCF7 and CAFs (previously transfected with control shRNA or shGPER plasmids) 3D co-culture spheroids (a single spheroid/well) grown for 3 days on agar-coated plates in the presence or absence of palbociclib (Palb). Scale bar 1000 μm. **D** Quantification of spheroid area; values of vehicle-treated spheroids were set as 100% upon which the area of palbociclib-treated spheroids was determined. Values represent the mean ± SD of three independent experiments performed in triplicate. (*) indicates *p* < 0.05

It has been demonstrated that CAFs are involved in the tumor resistance to chemotherapeutic agents, endocrine therapy and targeted treatments [50, 51]. On these bases and in accordance with the capability of CAFs to contribute to the acquisition of BC aggressive traits [50], we aimed to evaluate whether CAFs are implicated in the reduced responsiveness of BC cells to palbociclib treatment. To this end, we developed 2D and 3D co-culture assays that were optimized to quantify cell viability (Fig. 7A). In particular, co-cultures of MCF7 or T47D cells and CAFs, previously silenced or not for the expression of GPER, were treated with palbociclib. Then, the cell number and the spheroid area of MCF7 were measured, respectively, in 2D co-cultures (Fig. 7B; Additional File 6A) and 3D co-culture spheroid assays (Fig. 7C-D; Additional File 6B-C). Interestingly, palbociclib treatment reduced the viability only in MCF7 and T47D cells co-cultured with CAFs silenced for GPER expression (Fig. 7B-D; Additional File 6A-C). Taken together, these findings suggest that palbociclib prompts the regulation of pro-inflammatory mediators in CAFs via GPER, which is engaged in the functional interaction between BC cells and these main components of the tumor stroma toward a reduced palbociclib sensitivity.

## Discussion

Breast tumors are characterized by a high heterogeneity that influences both the response to treatments and the malignant features [89, 90]. Therefore, an accurate stratification of BC patients is crucial in order to achieve better clinical outcomes [89]. Advances in molecular profiling techniques have enabled the identification of different BC subtypes, which can be mainly defined by the expression of hormone receptors (ER and PR) and HER2 [91]. endocrine therapy is the traditional standard of care for the treatment of early and advanced-stage ER-positive BC [92, 93]. Although endocrine therapy used alone have demonstrated the capability to extend the survival rates of BC patients, a third of early-stage ER-positive BC may encounter resistance to endocrine therapy [94]. Thus, the identification of novel pharmacological strategies, including endocrine therapy, represents a crucial need toward better outcomes in BC [95]. In this context, the CDK4/6 inhibitors emerged as main therapeutics in the management of ER-positive metastatic BC [96]. The CDK4/6-Rb-E2F axis, which is fundamental for the transition of the cell cycle from the G1 to the S phase, is frequently dysregulated in cancer [21, 97]. Upon mitogenic signals, CDK4 and CDK6 lead to increased levels of cyclin D1, facilitating in this way the phosphorylation of the tumor suppressor Rb and the release of the transcription factor E2F [21], which up-regulates cyclins E1 and E2 toward their binding and activation of CDK2 and the consequent entry into S-phase of the cell cycle [21]. To date, the FDA approval of the use of CDK4/6 inhibitors like palbociclib, abemaciclib and ribociclib paved the way for new therapeutic options in ER-positive and HER2-negative advanced BC [98]. In particular, PALOMA-1 and PALOMA-2 clinical trials led to the FDA approval of palbociclib as the first CDK4/6 inhibitor in combination with letrozole for the first-line treatment of postmenopausal women with ER-positive and HER2-negative advanced BC [23, 24]. Subsequently, the results obtained from the PALOMA-3 trial led to the approval for the use of palbociclib together with fulvestrant in ER-positive and HER2-negative BC patients sensitive to endocrine therapy [99]. Nevertheless, about 30% of these patients do exhibit intrinsic resistance to all CDK4/6 inhibitors, including palbociclib, as well as acquired resistance [100]. Accordingly, the PALLAS and PENELOPE-B clinical trials failed to demonstrate the long-term efficacy of palbociclib in combination with endocrine therapy in patients with early-stage or residual high-risk invasive BC [30, 101]. In this scenario, it is currently under evaluation the clinical response to palbociclib combined with diverse therapeutics like the EGFR inhibitor cetuximab (NCT05226871), the PD-L1 antibody avelumab (NCT03147287), the Src/Abl kinase inhibitor bosutinib (NCT03854903), the BCL2 inhibitor venetoclax (NCT03900884) and chemotherapeutics (NCT03609047). Furthermore, several trials are underway to test the anticancer efficacy of palbociclib in association with novel SERDs, such as AZD9833 (NCT04711252 and NCT04964934) or giredestrant (NCT04546009).

Emerging data may shed light on the genetic alterations and the molecular events leading to palbociclib resistance in BC cells. For instance, increased expression levels of CDK4, CDK6, cyclin D1, cyclin E1 and cyclin E2 along with a reduction of *RB1* copy number were found associated with the resistance to palbociclib in ER-positive BC cells models [33, 36, 102, 103]. However, a deep comprehension of the mechanisms involved in BC resistance to palbociclib remains to be fully understood. In the present study, having established ER-positive BC cells resistant to palbociclib, we first explored the regulation of well-known mediators of estrogenic signaling like ERα and GPER. In this regard, we have ascertained a reduced ERα expression in these cells respect to the sensitive counterparts, in accordance with previous investigations [65–67]. Of note, the resistance to palbociclib has been reported to alter the ERα genome wide-binding pattern, leading to a decreased transcription of estrogen-regulated genes and a reduced sensitivity to the ER antagonists fulvestrant and tamoxifen [36, 102]. Furthermore, the loss of ER expression has been observed in ER-positive breast cancer patients resistant to CDK4/6 inhibitors [102]. Together, the down-regulation of ERα expression and transcriptional activity might have relevant implications for the outcomes of ER-positive breast cancer patients that display a resistance to the treatment with CDK4/6 inhibitors.

Several evidences have indicated that GPER mediates estrogenic signaling, thus contributing to the progression of breast tumors [10, 15]. In this regard, we have assessed that GPER expression increases in palbociclib-resistant compared with palbociclib-sensitive BC cells. In order to disclose the mechanisms involved in this effect, we focused on EGFR as a potential regulator of GPER transcription. Of note, EGFR silencing blunted the levels of GPER. In agreement with previous reports displaying an EGFR action as a transcription factor [71, 76, 77], ChIP assays revealed that EGFR can be recruited to the AT-rich site located within the promoter sequence of GPER in palbociclib-resistant BC cells. Moreover, we found that in these cells the increased expression of certain GPER-target genes, named c-Fos, EGR1 and CYR61, is abolished silencing GPER or EGFR expression. As a biological counterpart, we demonstrated that both EGFR and GPER are required to sustain the proliferative rate of palbociclib-resistant BC cells. In agreement with these findings and in line with previous data indicating a correlation between EGFR and GPER expression in BC [19], our bioinformatics analysis indicated that ER-positive BC patients characterized by high levels of both receptors exhibit a poor prognosis. Accordingly, the correlation of EGFR and GPER levels with clinical outcomes and aggressive features of BC, including the development of distant metastases and tumor size, has been assessed [20, 104–109]. Moreover, the expression levels of EGFR and GPER have been suggested as unfavorable predictors of survival in BC patients treated with tamoxifen [19, 110, 111]. These data well align with in vitro and in vivo studies indicating a main contribution of the EGFR/GPER-mediated signaling pathway in pro-tumorigenic features as well as drug resistance in BC cells [10, 18, 112, 113].

Several studies have indicated that cancer occurrence and progression greatly depend on the surrounding TME [114, 115]. In this regard, diverse pharmacological approaches targeting the most abundant cell type in the TME, namely the fibroblasts, have been evaluated and some have progressed to the preclinical phase [116]. Considering the high heterogeneity of CAFs, great efforts have been made to profile their populations [38, 117–119]. A better comprehension of this heterogeneity has been achieved through single-cell sequencing technologies, enabling the classification of CAFs into distinct subtypes including among others myofibroblastic (my)CAFs, inflammatory (i)CAFs and antigen-presenting (ap)CAFs [117]. In particular, iCAFs are characterized by reduced levels of α smooth muscle actin, elevated proliferative rates and high expression of inflammatory genes like cytokines and chemokines [120]. In this context, numerous studies have proposed a role for GPER in mediating the secretion by CAFs of pro-inflammatory mediators toward enhanced motile features in BC cells [13, 14]. To appreciate the mechanisms underlying a potential correlation of palbociclib resistance with GPER action within the TME, here we have shown that in breast CAFs palbociclib triggers main molecular sensors of GPER signaling, such as ERK activation and c-Fos, EGR1 and Cyr61 expression, as well as stimulates the expression of pro-inflammatory genes in a GPER-dependent manner. Furthermore, our bioinformatics analyses have shown that the 15 pro-inflammatory genes up-regulated by palbociclib through GPER in CAFs are associated with poor clinical features in ER-positive BC patients. On the basis of these observations, we evaluated whether CAFs may be involved in the sensitivity of BC cells to palbociclib. Of note, this CDK4/6 inhibitor was less effective when BC cells were co-cultured with CAFs, in accordance with previous findings indicating that fibroblasts may alter tumor responses to chemotherapeutics [52]. Meanwhile, BC sensitivity to palbociclib was rescued when BC cells were co-cultured with GPER-silenced CAFs. Overall, our findings suggest that GPER is involved in the regulation of pro-inflammatory genes within the TME upon palbociclib exposure, thus providing a rationale for further studies on the mechanisms mediating the resistance to palbociclib in BC.

## Conclusions

Our results provide novel insights into the molecular events that occur in the resistance to palbociclib in BC. In particular, our data suggest that GPER is up-regulated in an EGFR-dependent manner in palbociclib-resistant BC cells, therefore contributing to the insensitivity of BC cells to this CDK4/6 inhibitor. Furthermore, GPER activation by palbociclib triggered in CAFs the up-regulation of 15 pro-inflammatory genes correlated with poor outcomes in ER-positive BC patients. Further studies are warranted to better dissect the action of GPER within the breast TME in order to establish more comprehensive therapeutic options in BC patients resistant to palbociclib treatment.

### Supplementary Information

Additional file 1. Distinct expression profiles of the GPER-regulated inflammatory genes in the two k-means clusters. (A) Calculation of the optimal number of clusters k over a range of possible values as determined by the Silhouette Method. (B) Multiple boxplot showing the differential expression of the 15 pro-inflammatory genes in the two clusters obtained. (****) and (***) indicate *p*< 0.001 and *p* < 0.0001, respectively,“ns” indicates non-significant.

Additional file 2. Validation and molecular characterization of palbociclib-resistant T47D (T47D/PalbR) cells. (A) Representative pictures of spheroids (a single spheroid/well) from the T47D and T47D/PalbR spheroid cultures grown on agar-coated plates and exposed for 6 days to vehicle or 1 μM palbociclib (Palb), as indicated. Scale bar: 500 μm. (B) Quantification of spheroid growth; values of vehicle-treated T47D cells were set as 100% upon which spheroid growth was determined. (C) Immunoblot of ERα in T47D and T47D/PalbR cells. (D) Immunoblots of GPER and EGFR in T47D and T47D/PalbR cells. (E) GPER and EGFR protein expression in T47D and T47D/PalbR cells transiently transfected with a control shRNA or a shEGFR plasmid. Side panels show densitometric analyses of the blots normalized to β-actin, which served as loading control. Values represent the mean ± SD of three independent experiments performed in triplicate. (*) indicates *p* < 0.05.

Additional file 3. Cytoplasmic and nuclear levels of GPER in MCF7/PalbR respect to MCF7 cells.Immunoblots of cytoplasmic and nuclear fraction lysates derived from MCF7 cells and MCF7/PalbR cells. Side panel shows densitometric analysis of the blots normalized to lamin B1, which served as a nuclear marker. β-actin served as a cytoplasmic marker. Values represent the mean ± SD of three independent experiments performed in triplicate. (*) indicates *p* < 0.05.

Additional file 4. EGFR or GPER silencing restore palbociclib sensitivity in T47D/PalbR cells. (A) Representative pictures of spheroids (a single spheroid/well) from the T47D/PalbR/shRNA and T47D/PalbR/shEGFR spheroid cultures grown for 6 days on agar-coated plates. (B) Quantification of spheroid growth; values of T47D/PalbR/shRNA cells were set as 100%, upon which the number of T47D/PalbR/shEGFR cells was determined. (C) Efficacy of EGFR silencing in T47D/PalbR/shEGFR cells. (D) Representative pictures of spheroids (a single spheroid/well) from the T47D/PalbR/shRNA and T47D/PalbR/shGPER spheroid cultures grown for 6 days on agar-coated plates. Scale bar 500 μm. (E) Quantification of spheroid growth; values of T47D/PalbR/shRNA cells were set as 100% upon which the number of T47D/PalbR/shGPER cells was determined. (F) Efficacy of GPER silencing in T47D/PalbR/shGPER cells. (G) Colony formation assay in T47D/PalbR/shRNA and T47D/PalbR/shGPER cells. Plates were stained with Crystal Violet and colonies were counted following 10 days of incubation (H). (I) Protein levels of cyclin D1, cyclin E1 and GPER in T47D/PalbR/shRNA and T47D/PalbR/shGPER cells. Side panels show densitometric analyses of the blots normalized to β-actin, which served as loading control. Values represent the mean ± SD of three independent experiments performed in triplicate. (*) indicates *p* < 0.05.

Additional file 5. Palbociclib triggers the up-regulation of EGR1 and Cyr61 in CAFs through GPER. (A) Protein levels of EGR1 and Cyr61 in CAFs transiently transfected with a control shRNA or a shGPER plasmid and thereafter exposed for 4 h to vehicle (–) or 1 μM palbociclib (Palb). (B) Efficacy of GPER silencing. Side panels show densitometric analyses of the blots normalized to β-actin that served as loading control. Values represent the mean ± SD of three independent experiments performed in triplicate. (*) indicates *p* < 0.05.

Additional file 6. Palbociclib-treated T47D cells show a high survival rate following GPER activation in CAFs. (A) Viability of T47D cells (previously stained with CellTracker™CM-DiI dye) after 3 days treatment with vehicle or 1 μM palbociclib (Palb) and 2D co-cultured with CAFs that were previously transfected with control shRNA or shGPER plasmids and stained with CellTracker™ Green CMFDA dye. Values of vehicle-treated T47D cells were set as 100% upon which cell viability was determined. (B) Representative pictures of T47D and CAFs (previously transfected with control shRNA or shGPER plasmids) 3D co-culture spheroids (a single spheroid/well) grown for 3 days on agar-coated plates in the presence or absence of palbociclib (Palb). Scale bar 1000 μm. (C) Quantification of spheroid area; values of vehicle-treated spheroids were set as 100% upon which the area of palbociclib-treated spheroids was determined. Values represent the mean ± SD of three independent experiments performed in triplicate. (*) indicates *p* < 0.05.

## Data Availability

All data that were generated or analyzed during our study have been included in this article. Materials, additional data and protocols described within the manuscript will be made available from the authors upon reasonable request.

## Abbreviations

ACTB: Actin beta
AI: Aromatase inhibitor
BC: Breast cancer
CAFs: Cancer-associated fibroblasts
CDK: Cyclin dependent kinase
ChIP: Chromatin Immunoprecipitation
DMEM/F12: Dulbecco’s modified Eagle’s medium
DMSO: Dimethyl sulfoxide
E2: 17β-Estradiol
ECM: Extracellular Matrix
EGFR: Epidermal Growth Factor Receptor
EMA: European Medicines Agency
ER: Estrogen receptor
FBS: Fetal bovine serum
FDA: Food and Drug Administration
FGFR: Fibroblast Growth Factor Receptor
GPER: G protein estrogen receptor
HER2: Human Epidermal Growth Factor Receptor 2
IL-1β: Interleukin 1β
METABRIC: Molecular Taxonomy of Breast Cancer International Consortium
NPI: Nottingham Prognostic Index
OS: Overall survival
PI: Propidium iodide
PIK3CA: Phosphatidylinositol-4,5-Bisphosphate 3-Kinase Catalytic Subunit Alpha
PR: Progesterone receptor
Rb: Retinoblastoma protein
RFS: Relapse free survival
TCGA: The Cancer Genome Atlas
TME: Tumor microenvironment
TNBC: Triple negative breast cancer
