# Supplementary figures and images for: The G Protein Estrogen Receptor (GPER) is involved in the resistance to the CDK4/6 inhibitor palbociclib in breast cancer
Source: J Exp Clin Cancer Res. 2024 Jun 18;43:171. doi: 10.1186/s13046-024-03096-7 (PMC11184778; doi:10.1186/s13046-024-03096-7)

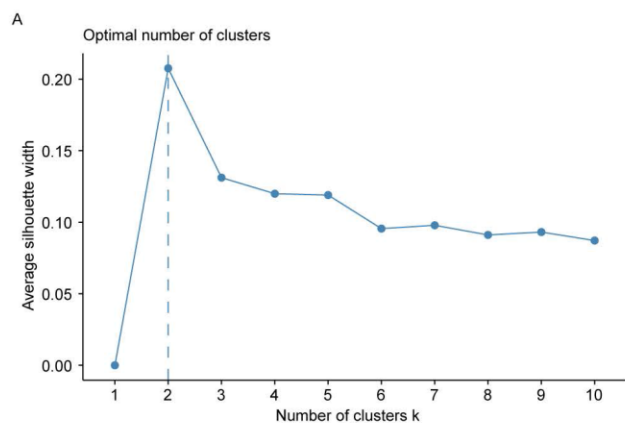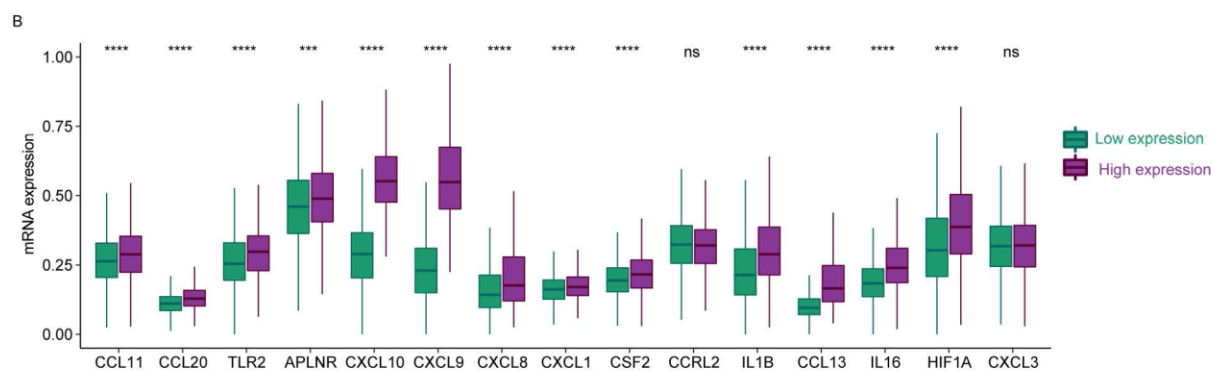

Supplement: Supplementary file 1 — Additional file 1. Distinct expression profiles of the GPER-regulated inflammatory genes in the two k-means clusters. (A) Calculation of the optimal number of clusters k over a range of possible values as determined by the Silhouette Method. (B) Multiple boxplot showing the differential expression of the 15 pro-inflammatory genes in the two clusters obtained. (****) and (***) indicate p< 0.001 and p < 0.0001, respectively,“ns” indicates non-significant. [file 13046_2024_3096_MOESM1_ESM.pdf]

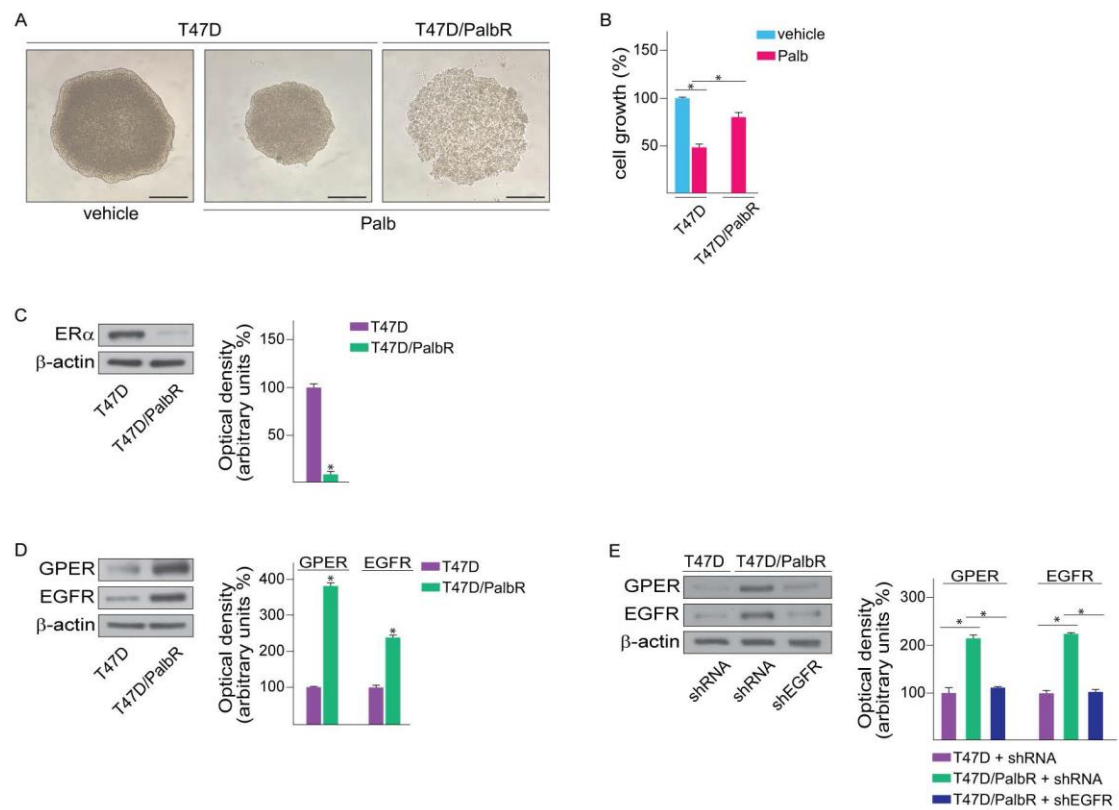

Additional File 2

Supplement: Supplementary file 2 — Additional file 2. Validation and molecular characterization of palbociclib-resistant T47D (T47D/PalbR) cells. (A) Representative pictures of spheroids (a single spheroid/well) from the T47D and T47D/PalbR spheroid cultures grown on agar-coated plates and exposed for 6 days to vehicle or 1 μM palbociclib (Palb), as indicated. Scale bar: 500 μm. (B) Quantification of spheroid growth; values of vehicle-treated T47D cells were set as 100% upon which spheroid growth was determined. (C) Immunoblot of ERα in T47D and T47D/PalbR cells. (D) Immunoblots of GPER and EGFR in T47D and T47D/PalbR cells. (E) GPER and EGFR protein expression in T47D and T47D/PalbR cells transiently transfected with a control shRNA or a shEGFR plasmid. Side panels show densitometric analyses of the blots normalized to β-actin, which served as loading control. Values represent the mean ± SD of three independent experiments performed in triplicate. (*) indicates p < 0.05. [file 13046_2024_3096_MOESM2_ESM.pdf]

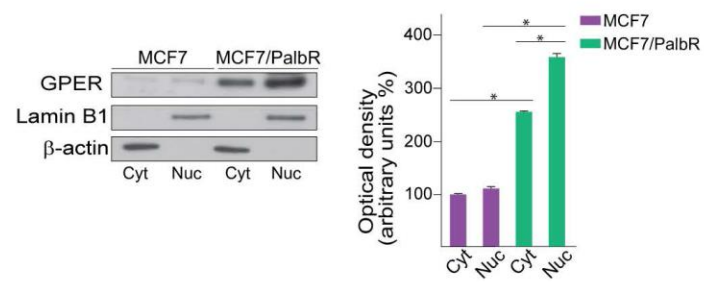

Additional File 3

Supplement: Supplementary file 3 — Additional file 3. Cytoplasmic and nuclear levels of GPER in MCF7/PalbR respect to MCF7 cells.Immunoblots of cytoplasmic and nuclear fraction lysates derived from MCF7 cells and MCF7/PalbR cells. Side panel shows densitometric analysis of the blots normalized to lamin B1, which served as a nuclear marker. β-actin served as a cytoplasmic marker. Values represent the mean ± SD of three independent experiments performed in triplicate. (*) indicates p < 0.05. [file 13046_2024_3096_MOESM3_ESM.pdf]

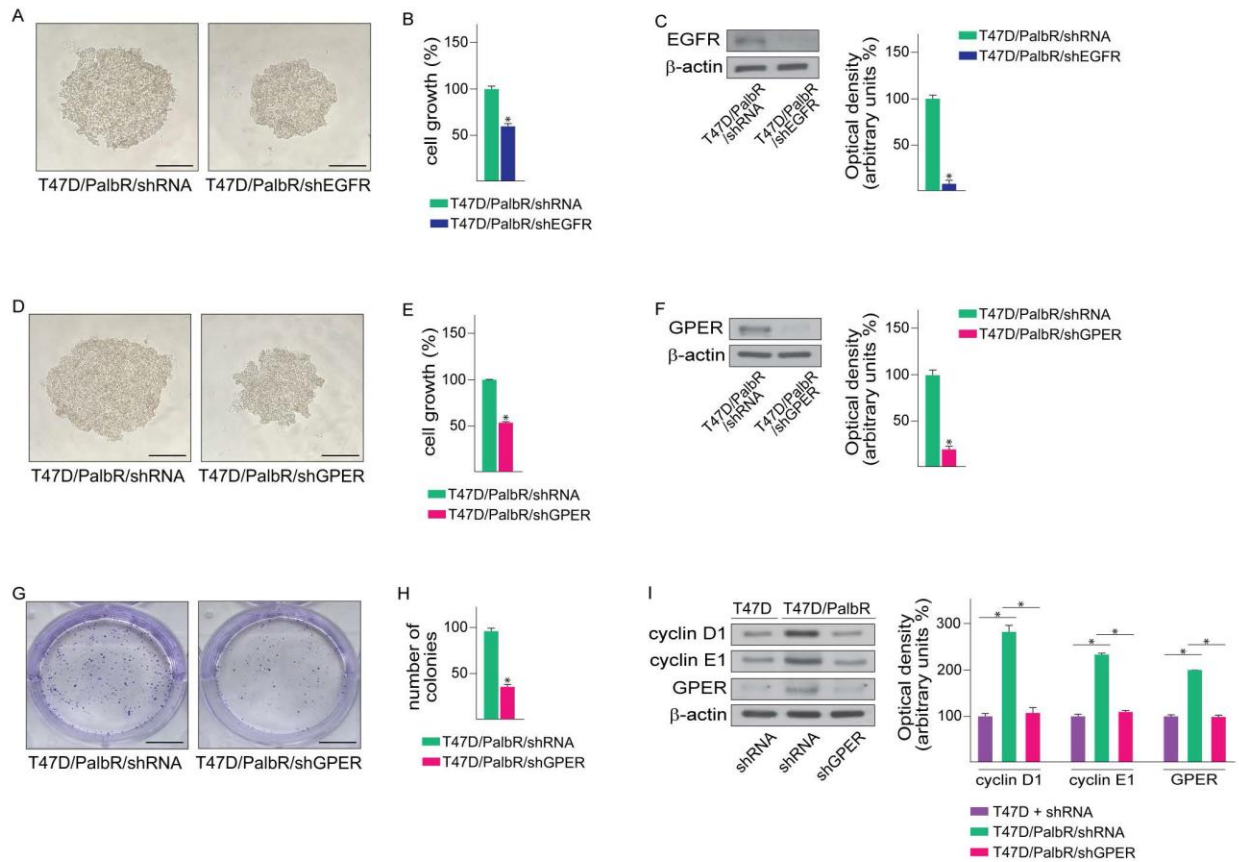

Supplement: Supplementary file 4 — Additional file 4. EGFR or GPER silencing restore palbociclib sensitivity in T47D/PalbR cells. (A) Representative pictures of spheroids (a single spheroid/well) from the T47D/PalbR/shRNA and T47D/PalbR/shEGFR spheroid cultures grown for 6 days on agar-coated plates. (B) Quantification of spheroid growth; values of T47D/PalbR/shRNA cells were set as 100%, upon which the number of T47D/PalbR/shEGFR cells was determined. (C) Efficacy of EGFR silencing in T47D/PalbR/shEGFR cells. (D) Representative pictures of spheroids (a single spheroid/well) from the T47D/PalbR/shRNA and T47D/PalbR/shGPER spheroid cultures grown for 6 days on agar-coated plates. Scale bar 500 μm. (E) Quantification of spheroid growth; values of T47D/PalbR/shRNA cells were set as 100% upon which the number of T47D/PalbR/shGPER cells was determined. (F) Efficacy of GPER silencing in T47D/PalbR/shGPER cells. (G) Colony formation assay in T47D/PalbR/shRNA and T47D/PalbR/shGPER cells. Plates were stained with Crystal Violet and colonies were counted following 10 days of incubation (H). (I) Protein levels of cyclin D1, cyclin E1 and GPER in T47D/PalbR/shRNA and T47D/PalbR/shGPER cells. Side panels show densitometric analyses of the blots normalized to β-actin, which served as loading control. Values represent the mean ± SD of three independent experiments performed in triplicate. (*) indicates p < 0.05. [file 13046_2024_3096_MOESM4_ESM.pdf]

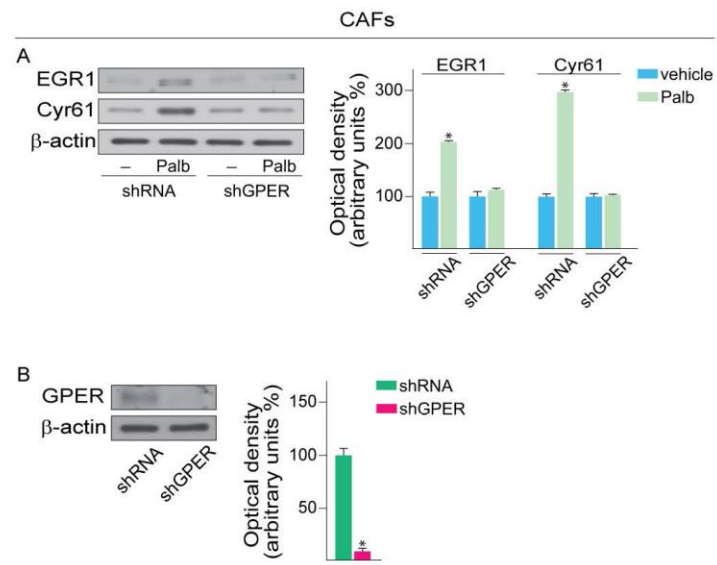

Supplement: Supplementary file 5 — Additional file 5. Palbociclib triggers the up-regulation of EGR1 and Cyr61 in CAFs through GPER. (A) Protein levels of EGR1 and Cyr61 in CAFs transiently transfected with a control shRNA or a shGPER plasmid and thereafter exposed for 4 h to vehicle (–) or 1 μM palbociclib (Palb). (B) Efficacy of GPER silencing. Side panels show densitometric analyses of the blots normalized to β-actin that served as loading control. Values represent the mean ± SD of three independent experiments performed in triplicate. (*) indicates p < 0.05. [file 13046_2024_3096_MOESM5_ESM.pdf]

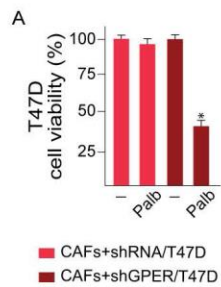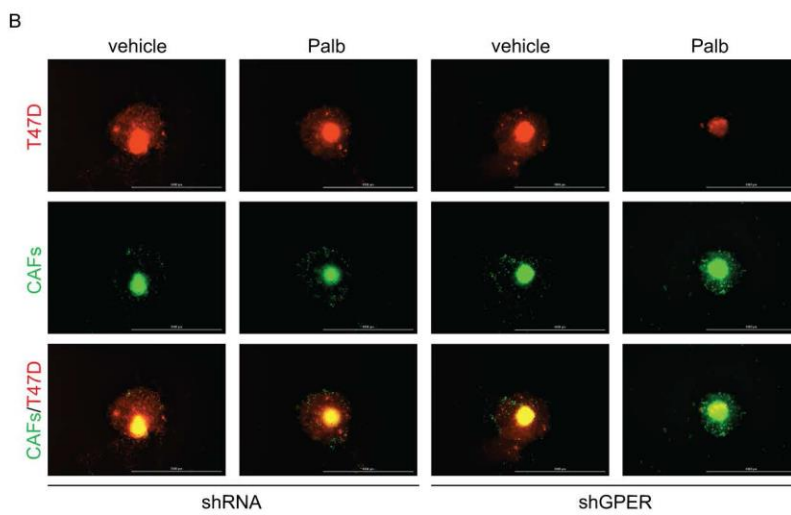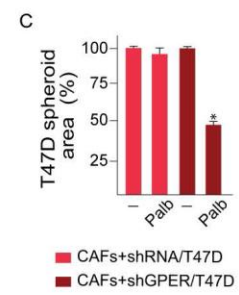

Supplement: Supplementary file 6 — Additional file 6. Palbociclib-treated T47D cells show a high survival rate following GPER activation in CAFs. (A) Viability of T47D cells (previously stained with CellTracker™CM-DiI dye) after 3 days treatment with vehicle or 1 μM palbociclib (Palb) and 2D co-cultured with CAFs that were previously transfected with control shRNA or shGPER plasmids and stained with CellTracker™ Green CMFDA dye. Values of vehicle-treated T47D cells were set as 100% upon which cell viability was determined. (B) Representative pictures of T47D and CAFs (previously transfected with control shRNA or shGPER plasmids) 3D co-culture spheroids (a single spheroid/well) grown for 3 days on agar-coated plates in the presence or absence of palbociclib (Palb). Scale bar 1000 μm. (C) Quantification of spheroid area; values of vehicle-treated spheroids were set as 100% upon which the area of palbociclib-treated spheroids was determined. Values represent the mean ± SD of three independent experiments performed in triplicate. (*) indicates p < 0.05. [file 13046_2024_3096_MOESM6_ESM.pdf]
